# Evaluation of a New Methylimidazole‐Containing Thiosemicarbazone as a Cu^+^/Cu^2+^‐Targeting Ligand in the Context of Alzheimer's Disease

**DOI:** 10.1002/chem.202502754

**Published:** 2025-11-28

**Authors:** Barbara Marinho Barbosa, Charlène Esmieu, Antal Galvácsi, Mariana Viana Costa, Adèle Brison, Sonia M. Ladeira, Jade de Oliveira, Csilla Kállay, Christelle Hureau, Nicolás A. Rey

**Affiliations:** ^1^ Department of Chemistry Pontifical Catholic University of Rio de Janeiro (PUC‐Rio) Rio de Janeiro Brazil; ^2^ Laboratoire De Chimie de Coordination (LCC) CNRS, University of Toulouse Toulouse France; ^3^ Department of Inorganic and Analytical Chemistry University of Debrecen Debrecen Hungary; ^4^ Department of Biochemistry Basic Health Sciences Institute Federal University of Rio Grande Do Sul Rio Grande do Sul Brazil

**Keywords:** Alzheimer's disease, AÎ^2^ aggregation, Copper, ROS, Thiosemicarbazone

## Abstract

The binding of copper ions to amyloid‐β (Aβ) peptide leads to reactive oxygen species (ROS) formation and toxic soluble oligomers, contributing to oxidative stress in Alzheimer's disease (AD). Thus, studying compounds with moderate copper affinity is a promising strategy to prevent its interaction with Aβ and reduce toxicity. Here, we evaluated a new tri‐coordinating thiosemicarbazone (**HXE**) with chelating properties to regulate cuprotoxicity in AD. The ligand was nontoxic against HT‐22 hippocampal neuronal cells and bound Cu^+^ and Cu^2+^ at pH 7.4, with affinity constants (log *K*_cond_) of 8.7 and 12.3, respectively, showing high selectivity over Zn^2+^ (log *K*_app_ = 5.0). In the presence of Aβ and Cu^2+^, **HXE** formed stable ternary complexes at physiological pH. Ascorbate consumption and coumarin‐3‐carboxylic acid fluorescence assays showed that the ligand significantly reduces Cu(Aβ_16_)‐mediated ROS production. It also prevented Cu^2+^‐induced modulation of Aβ_40_ self‐assembly and restored the typical fibrillar structure of apo‐Aβ_40_ aggregates. Overall, **HXE** effectively modulates metal‐associated Aβ toxicity and emerges as a promising candidate for AD bioinorganic management.

## Graphical abstract

**HXE** modulates Cu(Aβ) interaction, reducing ROS production and shifting peptide aggregation in the presence of Cu from toxic amorphous aggregates to less harmful fibrillar structures. These findings highlight **HXE** potential to interfere with Cu‐induced oxidative stress and aggregation pathways relevant to AD.

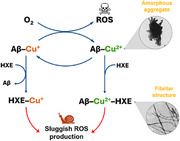

## 1 Introduction

Copper is an essential trace element in biological systems, being required as a structural component, electron transporter agent and cofactor of redox enzymes, including Cu/Zn superoxide dismutase (SOD1) or cytochrome c oxidase, that play a critical role in antioxidant defense and mitochondrial respiratory chain, respectively [1, 2]. In the nervous system, this metal actively participates in neurotransmitter biosynthesis, cognitive processes and gene expression [3]. Copper homeostasis in the brain is finely regulated by the action of transporters, enzymes and chaperones to ensure the proper distribution and avoid toxic accumulation [4, 5, 6]. Copper can be found either inertly bound to ceruloplasmin (Cp), representing 75–95% of the total amount, or as “non‐Cp‐Cu” (“free copper”) [7], when bound to coordinating biomolecules, such as Human Serum Albumin (HSA) that provides a higher lability and bioavailability of the metal [4, 8, 9]. The passage of this metal through the blood‐brain barrier (BBB) is a highly selective process and depends on transporters such as CTR1, ATP7A, and ATP7B [4]. Disturbance in its homeostasis has been linked to several pathological conditions, as neurodegenerative disorders, cancer and cardiovascular diseases [4, 10].

In Alzheimer's disease (AD), growing evidence points to physiological metal ions (mainly Cu, Zn and Fe) imbalance as the main bioinorganic factor linked to this pathology's progression [7, 11, 12, 13]. Copper has been found to be present at high concentrations in senile plaques, one of the main hallmarks of the disease [14, 15, 16]. These insoluble deposits are mainly composed by aggregates of a 40–42 amino acid residues peptide, called amyloid‐β (Aβ), derived from the pathological cleavage of the transmembrane amyloid precursor protein (APP) [17, 18, 19, 20, 21]. At pH around 7, Aβ forms stable complexes with Cu ions, and this interaction modulates the peptide's aggregation kinetics, shifting the equilibrium from the formation of insoluble fibrils to toxic soluble oligomers [22, 23, 24, 25, 26]. Furthermore, the Cu(Aβ) complex has a Cu‐associated redox activity, cycling between Cu^+^ and Cu^2+^ states in the presence of molecular oxygen and biological reductants, such as ascorbate. This process culminates in the generation of reactive oxygen species (ROS), which contribute to neuronal oxidative stress, a central mechanism implicated in neurodegeneration [27, 28].

Therefore, the use of ligands (including some of the multitarget type) has emerged as a promising strategy to limit the interaction of Aβ with copper, relieving the toxicity mediated by Cu(Aβ) [5, 29, 30, 31, 32, 33, 34, 35, 36, 37, 38, 39, 40]. However, to date, no chelating agent has shown satisfactory results in clinical trials, not even those that showed satisfactory *in vitro* results [41, 42]. It is worth noting that most of the proposed compounds so far have been designed to specifically target Cu^2+^, not taking into consideration the crucial role of redox transition to Cu^+^ for the toxicity of the Cu(Aβ) complex [5]. At physiological pH, Cu^2+^ ions bind to Aβ with a square‐planar geometry, through the imidazole (Im) rings of two His residues (His6 and either His13 or His14), the N‐terminal amine and the carboxylate group of Asp1, with an affinity of 10^9^ – 10^10^ M^‒1^ [43, 44, 45, 46]. In turn, Cu^+^ is linearly coordinated to the peptide, involving two Im groups of His residues, with an affinity in the literature ranging from 10^7^ to 10^10^ M^‒1^ [46, 47, 48, 49]. Thus, chelators that focus on both oxidative states of copper may be an attractive approach to interrupt the pathological redox cycles involved in Aβ neurotoxicity [50]. Besides, ligands that target Cu^2+^ face a challenge related to selectivity toward other metal ions present in the extracellular medium, mainly Zn^2+^, that displays a similar preference for N/O‐donors and is present at a higher concentration than copper in the synaptic cleft, reducing the compounds’ efficacy [42, 51]. The difference in the coordination chemistry of the metal ions can be explored to design an improved chelator and overcome selectivity limitations. Cu^2+^ and Zn^2+^ are considered borderline acids based on Pearson's concept [52], showing a preference for intermediate bases, as those containing delocalized N/O‐donors. However, due to the Jahn‐Teller effect, copper tends to adopt square‐planar or distorted octahedral geometries, whereas zinc has no electronic constraints and is thus usually found tetrahedrally coordinated. With respect to Cu^+^, it has a softer character and preferably binds to soft donors, such as sulfur, in linear, trigonal planar or tetrahedral geometries.

Recently, some of us have shown interest in targeting Cu^+^ ions in the context of AD [50, 53, 54, 55, 56]. Those compounds prove to be effective in removing copper from Aβ, lessening or stopping Cu(Aβ)‐induced ROS production and showing satisfactory selectivity toward Zn^2+^ [54, 56], encouraging the use of this strategy in the rational design of novel potential chelating agents for AD therapy. To be considered a promising drug candidate, a compound must meet several criteria: (i) ability to interrupt Cu(Aβ) redox cycle, (ii) rapid coordination kinetics [57, 58], (iii) capacity to abstract Cu^+^/Cu^2+^ from Aβ or forming stable compound‐Cu‐Aβ ternary complexes without compromising copper ions involved in physiological activities, and (iv) high selectivity over other metal ions abundant in the extracellular environment. Additionally, favorable pharmacokinetic properties, especially regarding toxicity and permeability through the BBB, are important criteria to fulfil if medical applications are foreseen [42].

Over the past years, some of us have extensively investigated *N*‐acylhydrazones (NAHs) as a promising class for the management of metal‐enhanced aggregopathies [59, 60, 61, 62, 63, 64]. NAHs have demonstrated suitable affinity for Cu^2^⁺ and Zn^2^⁺, being able to compete with amyloidogenic peptides without disrupting metal ion homeostasis in the organs of healthy Wistar rats [62]. More recently, a new family of NAHs derived from 1‐methylimidazole‐2‐carboxaldehyde has shown improved properties [59, 65, 66, 67, 68, 69]. The inclusion of this five‐membered nitrogen‐containing ring into previously studied NAH structures proved effective in enhancing water solubility, hydrolytic stability, and reducing toxicity, while also helping to complete the coordination sphere of Cu^2^⁺. Based on this, and aiming to target Cu⁺ ions in addition to Cu^2+^, we propose in this work the replacement of the hydrazone moiety by a thiosemicarbazone group, to introduce a sulfur atom at the coordination site—a softer donor atom.

**FIGURE 1:**
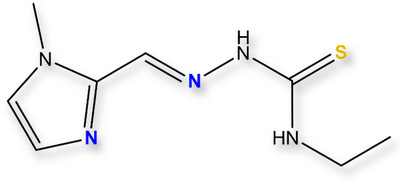
**HXE** structure. Potential donor atoms are highlighted in color.

Thiosemicarbazones (TSCs) and their metal complexes are known in the literature for their wide range of biological properties: anticancer, antioxidant, antiinflammatory, antibacterial, and antiviral activities [70, 71]. In AD context, previous studies showed that TSCs derivates have the ability to inhibit Aβ self or Cu‐induced aggregation, antioxidant activity, acetylcholinesterase (AChE) activity, and have low toxicity in cell models and a suitable permeability through BBB [72, 73, 74, 75, 76, 77, 78, 79, 80]. Hence, we herein report an investigation into the therapeutic potential of a new TSC–1‐methylimidazole‐2‐carboxaldehyde 4‐ethyl‐3‐thiosemicarbazone—(**HXE**, Figure 1) containing a methylimidazole moiety, which was designed to target both biologically relevant oxidation states of copper in the context of AD. **HXE** was evaluated regarding its affinity for Cu^2+^ and Cu^+^ ions, selectivity relative to Zn^2+^ and the ability to reverse deleterious effects induced by Cu(Aβ), including the generation of ROS and the metal‐induced modulation of peptide aggregation.

## 2 Materials and Methods

### 2.1 Chemicals

Solvents and reactants were purchased from commercial sources in the higher purity available. Fresh stock solutions of **HXE** (5 mM, Milli‐Q), ascorbate (5 mM, Milli‐Q) and 3‐CCA (2 mM, 200 mM phosphate buffer pH 7.4) were prepared at the beginning of each day. Stock solutions of Cu^2+^ and Zn^2+^ (0.1 M, water), from the salts CuSO_4_·5 H_2_O and ZnSO_4_·1 H_2_O, were used. BCA (bicinchoninic acid) (10 mM) was prepared by dissolution in Milli‐Q water and the exact concentration was determined by UV‐Vis titration with CuSO_4_ in the presence of 3 equivalents of ascorbate to obtain the Cu^+^ [Cu(BCA)_2_]^3‒^ complex (ε_562 nm_ = 8060 ± 30 M^−1^ cm^−1^, 50 mM HEPES pH 7.4). Tetrakis(acetonitrile)copper(I) tetrafluoroborate salt was bought from TCI. Stock solutions were prepared inside an argon flushed glove box by dissolving the powder in ACN or ACN‐*d*_3_ for NMR experiments. The concentration was determined by UV‐Vis titration with an excess of BCA in a 50 mM HEPES pH 7.4 solution. Stock solution of Thioflavin T (ThT) was prepared by dissolving the powder in Milli‐Q and concentration was determined by UV‐Vis (ε_412 nm_ = 33000 M^−1^ cm^−1^) [81].

GGH (sequence GGH‐NH_2_), Aβ_16_ (sequence DAEFRHDSGYEVHHQK‐NH_2_) and Aβ_40_ (sequence DAEFRHDSGYEVHHQKLVFFAEDVGSNKGAIIGLMVGGVV) peptides were bought from GeneCust or ApexBio with purity grade > 95%. Stock solutions of GGH and Aβ_16_ were prepared by the solubilization of the powder in Milli‐Q water. For GGH, the concentration was determined by UV‐Vis titration with Cu^2+^ until no change in *d*‐*d* band (530 nm) was observed. For Aβ_16_, the concentration of the stock was determined by the absorption of Tyr10 at 276 nm, corrected for the absorption at 300 nm (ε_276 nm_ = 1410 M^−1^ cm^−1^, pH ∼ 2) in UV‐Vis [82]. Aβ_40_ was monomerized and purified by FPLC before utilization following the previously reported protocol [83]. Concentration of the FPLC fractions of interest were determined in a 500 mM NaOH solution using the electronic absorption of deprotonated Tyr10 at 293 nm, corrected for the absorption at 360 nm (ε_293 nm_ = 2400 M^−1^ cm^−1^, pH >13) [82].

### 2.2 Instruments

Elemental analyses were performed in a Perkin Elmer 2400 Series II Flash Combustion Analyzer in duplicate at room temperature.

Infrared spectra were acquired on a Perkin Elmer FT‐IR Frontier and Perkin Elmer Spectrum Two FT‐IR spectrometers.

^1^H NMR spectra were recorded on a Bruker AvanceIII 400 or an Avance NEO 600 spectrometers at room temperature using dimethylsulfoxide (DMSO‐*d*_6_) or aqueous solution with 10% D_2_O as solvent. Suppression of the water signal was performed using excitation sculpting method. Crystallographic data of **HXE** and its Cu^2+^ complex were collected on a Rigaku XtaLAB Synergy Dualflex diffractometer using a PhotonJet X‐ray source (CuK α, *λ* = 1.54184 Å). An Oxford Cryosystems Cryostream cooling device was used to collect data at low temperature [100(2) K]. Potentiometric titrations were performed in a MOLSPIN pH‐meter equipped with a Metrohm 6.0234.100 combined glass electrode (Metrohm) in the pH range 2.5–11.5, while the dosing of the titrant was made with a computer‐controlled MOL‐ACS microburette.

EPR spectra were acquired using an Elexsys E‐500 Bruker spectrometer operating at a microwave frequency of approximatively 9.4 GHz with a modulation amplitude of 5G, 160 s of conversion time, 2 scans and 2500 to 3900 G as magnetic field range. The experiments were carried out at 120 K using a liquid nitrogen cryostat.

UV‐Vis experiments were performed in a Hewlett Packard Agilent 5484 spectrophotometer under controlled temperature and continuous stirring (25°C, 800 rpm) using a 1 cm cuvette or, alternatively, in a Spectrostar nano (BMG Labtech) spectrophotometer using a 96 wells plate (Greiner, F‐bottom, UV clear) at 25°C, stirring at 500 rpm for 10 s before each measurement. For cell viability experiments, a SpectraMax M5 spectrophotometer (Molecular Devices) was used.

Cyclic Voltammograms were acquired on an Autolab PGSTAT302N potentiostat controlled with GPES 4.9 software and using three electrodes: a glassy carbon electrode as working electrode, a platinum wire as counter electrode and a saturated calomel electrode (SCE) as reference.

Fluorescence experiments were performed in plates readers from BMG Labtech (ClarioStar, Omega, and Optima) using a 96 or 384 wells plate.

Eletronic Microscopy was performed in a Jeol JEM‐1400, JEOL inc, Pea‐body, MA, USA at 80 kV and images were taken with a digital camera (Ametek rio 9) at magnifications between 3000 and 12000.

### 2.3 Syntheses

***HXE*** ─1‐methylimidazole‐2‐carboxaldehyde 4‐ethyl‐3‐thiosemicarbazone (**HXE**) was synthesized through the base Schiff condensation between 1‐methylimidazole‐2‐carboxaldehyde (0,2212 g, 2.0 mmol) and 4‐ethyl‐3‐thiosemicarbazide (0.2390 g, 2.0 mmol). Both reactants were dissolved in the minimal volume of ethanol and the thiosemicarbazide solution was dropped over the aldehyde. Reaction was catalyzed with HCl, resulting in a white precipitate, which was filtered and washed with cold ethanol. The reactional scheme can be found in Figure S1. White crystals were obtained through the recrystallization of the powder in an 80/20 EtOH/H_2_O mixture.

White powder (C_8_H_14_N_5_SCl; **HXE**, HCl) Yield: 66%. M.W. = 247.75 g mol^−1^. Elemental analysis: Calculated ‒ C: 38.8%, H: 5.7%, N: 28.3%, S: 12.9%. Experimental ‒ C: 38.7%, H: 5.7%, N: 27.7%, S: 13.1%. Main IR bands (ATR, cm^−1^): ν(N‒H) 3213, 3162; ν(C = N)_azomethine_ 1607; ν(C = S) 805 (Figure S2).

^1^H NMR (DMSO‐*d*_6_; ppm): 1.18 (t, 3H), 3.60 (m, 2H), 3.88 (s, 3H), 7.73 (d, 1H, ^3^ *J* = 1.9 Hz), 7.76 (d, 1H, ^3^ *J* = 1.9 Hz), 8.01 (d, 1H), 9.28 (t, 1H), 12.14 (s, 1H).

***[Cu(XE)Cl]*** ─ 0.2 mmol of ligand and CuSO_4_ were solubilized in the least necessary volume of a mixture 80/20 EtOH/H_2_O. **HXE** solution, which showed an initial pH of 6, was adjusted to pH 8 and then dropwise added to the Cu^2+^ solution. The system was kept under stirring at 50°C for 1 h, resulting in a dark green solution with a green precipitate. The precipitate was separated by filtration and washed with cold ethanol. Part of the powder was then resolubilized in 80/20 EtOH/H_2_O and the solution was kept at room temperature for slow evaporation. Green needle crystals were obtained after two weeks.

Green powder (C_8_H_12_ClCuN_5_S). Yield: 28%. M.W. = 309.29 g mol^−1^. Main IR bands (ATR, cm^−1^): ν(N‒H) 3324; ν(C = N) _azomethine_ 1618; ν(C = S) 708 (Figure S2).

### 2.4 X‐Ray Diffraction

Omega scans were performed for data collection. An empirical absorption correction was applied and the structures were solved by intrinsic phasing method (ShelXT) [84]. All nonhydrogen atoms were refined anisotropically by means of least‐squares procedures on F^2^ with ShelXL. All the hydrogen atoms were refined isotropically at calculated positions using a riding model, except for the *N*‐bound hydrogen atoms, which were located in different Fourier maps and refined freely. Deposition Numbers <url href = “https://www.ccdc.cam.ac.uk/services/structures?id = https://doi.org/10.1002/chem.202502754”> 2477871 (for **HXE**) and 2477872 {for [Cu(**XE**)Cl]}</url> contain the supplementary crystallographic data for this paper. These data are provided free of charge by the joint Cambridge Crystallographic Data Centre and Fachinformationszentrum Karlsruhe <url href = “http://www.ccdc.cam.ac.uk/structures”>Access Structures service</url>.

### 2.5 In Silico Calculations

Some pharmacological parameters such as cLog P, cLog S, PSA, Druglikeness, and Drug Score were calculated for **HXE** using Osiris Property Explorer: DataWarrior, software freely available for download at http://www.organic‐chemistry.org/prog/peo/.

### 2.6 Toxicity Against HT‐22 Cells

***Cell Culture and Experimental Design*** ─HT‐22 cells (mouse hippocampal neuronal cell line) were cultured in Dulbecco's Modified Eagle's Medium (DMEM; Sigma‐Aldrich, D7777) supplemented with 10% fetal bovine serum (FBS; CRIPION, São Paulo, Brazil) and 100 IU mL^−1^ penicillin/streptomycin (Sigma‐Aldrich, P0781). Cells were maintained at 37 °C in a humidified atmosphere containing 5% CO_2_ and 95% air. HT‐22 cells were seeded at a density of 2 × 10⁴ cells cm^−2^ [85]. After 24 h, the cells were treated with HXE at concentrations of 5, 10, 20, 50, 100, or 200 µM for an additional 24 h.

***Cell Viability Assay*** ─Cell viability was assessed using the 3‐(4,5‐dimethylthiazol‐2‐yl)‐2,5‐diphenyltetrazolium bromide (MTT) assay [86]. After 24 h of exposure, neuronal cells were incubated with 0.5 mg mL^−1^ MTT (Sigma‐Aldrich) for 3 h at 37°C in a 5% CO_2_ atmosphere. Experiments were carried out in 96‐well plates, and the formazan crystals formed by the reduction of MTT were dissolved in DMSO. Formazan absorbance was measured at a wavelength of 540 nm. Results are expressed as the percentage of control cells [85, 86]. Statistical analysis was performed using one‐way ANOVA test.

### 2.7 Potentiometric Titrations

Stock solutions of CuCl_2_ and ZnCl_2_ were prepared from analytical grade reagents and their concentrations were checked gravimetrically via the precipitation of oxinates. The other stock solutions (KOH, HCl, KCl, potassium hydrogen phthalate) were also prepared from analytical grade reagents.

The concentration and the deprotonation constants of the ligand and the stability of the Cu^2+^ and Zn^2+^ complexes were determined by pH‐metric titration at 298.0 ± 0.1 K and at a constant ionic strength of 0.2 M KCl. All potentiometric measurements were carried out in 3.00 mL aqueous samples at 2 mM ligand concentration with a metal(II) ion‐to‐ligand ratio of 1:1. Titrations were performed using carbonate free stock solution of potassium hydroxide of accurately known concentration. During the experiments, argon was bubbled through the samples to ensure the absence of oxygen and carbon dioxide. The samples were stirred by a VELP Scientific magnetic stirrer. The recorded pH readings were converted to hydrogen ion concentration as described by Irving *et al.* [87]. Protonation constants of **HXE** and overall stability (log *β*_pqr_) constants of complexes were calculated by means of the general computational programs SUPERQUAD and PSEQUAD [88, 89], based on Equations (1) and (2):

(1) $$pM+qH+rL=M_{p}H_{q}L_{r}$$

(2) $$\beta_{pqr}=\frac{\left[M_{p}H_{q}L_{r}\right]}{{\left[M\right]}^{p}\cdot {\left[H\right]}^{q}\cdot {\left[L\right]}^{r}}$$

The concentration distribution curves were generated by the MEDUSA program using the protonation constants of the ligand and the stability constants of the Cu^2+^ and Zn^2+^ complexes at the same reactant concentrations as in the titrations [90].

### 2.8 UV‐Vis Titrations With Cu^2+^ and Zn^2+^

**HXE** solution was diluted with Milli‐Q to achieve a final concentration of 50 µM in HEPES (50 mM, pH 7.4). Substoichiometric (about 0.2 equiv. per **HXE**) ratio of Cu^2+^ or Zn^2+^ solutions were added to the ligand solution and the electronic absorption spectra were recorded after each addition. The apparent affinity constant (*K*_app_—in a buffered solution pH 7.4) for Zn^2+^ was determined according to Table 1 and Equation (5):

**TABLE 1:** Equilibrium for the formation of [Zn(**XE**)(H_2_O)_x_]^+^.

	HXE	+	Zn^2+^	⇌	[Zn(XE)(H_2_O)_x_]^+^
Initial:	$C_{0}$		$C_{1}$		0
Equilibrium:	$C_{0}-\alpha C_{0}$		$C_{1}-\alpha C_{0}$		$\alpha C_{0}$

(3) $$K_{app}=\frac{\alpha }{1-\alpha }\frac{1}{C_{1}-\alpha C_{0}}$$

(4) $$K_{\mathrm{app}}C_{0}\alpha^{2}-\left(K_{\mathrm{app}}C_{0}+K_{\mathrm{app}}C_{1}+1\right)\alpha +K_{\mathrm{app}}C_{1}=0$$

(5) $$\alpha =\frac{-b+\sqrt{b^{2}-4ac}}{2a}$$

$$\mathrm{For}:a=K_{app}C_{0}$$

$$b=-\left(K_{app}C_{0}+K_{app}C_{1}+1\right)$$

$$c=K_{app}C_{1}$$

### 2.9 Competition Assays

The affinity of the ligand for Cu^+^ and Cu^2+^ was determined by competition assays monitored by UV‐Vis spectroscopy, aiming to estimate the conditional affinity constants (*K*_cond_), in which the effects of the buffer are neglected.

***Affinity for Cu^2+^*** ─Mixtures of [Cu(**XE**)(H_2_O)]^+^ and the GGH peptide were prepared in a 50 mM HEPES pH 7.4 solution in a 96 wells plate, in triplicate, and the UV‐Vis spectra of the samples were acquired with an interval of 15 min. The experiment was performed by the addition of different equivalents of GGH to the [Cu(**XE**)(H_2_O)]^+^ solution. After the equilibrium shown in Equation (6) was reached, the progression of the reaction (i.e., α = [Cu(GGH)]/[Cu^2+^]_0_) was determined using the absorbance at 383 nm, related to the complex [Cu(**XE**)(H_2_O)]^+^, as demonstrated in Equation (7). Due to the presence of an excess of ligand in solution, the absence of free Cu^2+^ is assumed. The contribution of a ternary [Cu(**XE**)(Im_GGH_)]^+^ complex was not considered in the fitting model. Given that the experimental data were well described by including only the two binary species, we supposed that the formation of the ternary complex does not significantly impact the estimation of the affinity constant. OBS.: throughout the manuscript, for the ternary species involving GGH or Aβ, we consider the net charge of Im rather than those of the peptides themselves for matter of simplicity.

(6) $${\left[Cu\left(XE\right)\left(H_{2}O\right)\right]}^{+}+GGH⇌Cu\left(GGH\right)+HXE$$

The ratio between the conditional affinity of **HXE** and GGH for Cu^2+^ was calculated by fitting the experimental data with Equation (10) (Origin Software), where [Cu^2+^]_0_ = C_0_, [**HXE**]_0_ = C_1_, [GGH]_0_ = C_2_ and β is the stability constant for [Cu(**XE**)(H_2_O)]^+^.

(7) $$\alpha =1-\frac{Abs_{383nm}}{\varepsilon_{383nm}^{{\left[Cu\left(XE\right)\left(H_{2}O\right)\right]}^{+}}}\frac{1}{C_{0}}$$

(8) $$K=\;\;\frac{\beta }{K_{cond}^{HXE}}=\frac{\alpha }{1-\alpha }\frac{C_{1}-C_{0}+\alpha .C_{0}}{C_{2}-\alpha .C_{0}}$$

(9) $$\left(K-1\right)C_{0}\alpha^{2}-\left(KC_{0}+KC_{2}+C_{1}-C_{0}\right)\alpha +KC_{2}=0$$

(10) $$\alpha =\frac{-b-\sqrt{b^{2}-4ac}}{2a}$$

$$\mathrm{For}:a=\left(K-1\right)C_{0}$$

$$b=-\left(KC_{0}+KC_{2}+C_{1}-C_{0}\right)$$

$$c=KC_{2}$$

***Affinity for Cu^+^*** ─ Competition experiment between [Cu(BCA)_2_]^3‒^ and **HXE** was performed anaerobically in a 50 mM HEPES pH 7.4 solution by varying the concentration of **HXE** added. Solution of [Cu(BCA)_2_]^3‒^ was prepared inside the glove box and one equivalent of Na_2_S_2_O_4_ was used to avoid oxidation of the metal. **HXE** was then added with a Hamilton syringe into the sealed cuvette equipped with a screw cap. The apparent affinity constant for [Cu(**XE**)(H_2_O)] was determined by solving Equation (17) for each C_2_ value, where [Cu^+^]_0_ = C_0_, [BCA]_0_ = C_1,_ [**HXE**]_0_ = C_2_, and β_2_ is the stability constant for [Cu(BCA)_2_]^3‒^. The value reported corresponds to the mean and standard deviation between the different points.

(11) $${\left[\mathrm{Cu}{\left(\mathrm{BCA}\right)}_{2}\right]}^{3-}+\mathrm{HXE}⇌{\left[\mathrm{Cu}\left(\mathrm{XE}\right)\left(H_{2}O\right)\right]}^{+}+2\mathrm{BCA}$$

(12) $${\left[Cu{\left(BCA\right)}_{2}\right]}^{3-}=\alpha_{BCA}=\frac{Abs_{562nm}}{\varepsilon_{562nm}^{{\left[Cu{\left(BCA\right)}_{2}\right]}^{3-}}}$$

(13) $${\left[Cu\left(XE\right)\left(H_{2}O\right)\right]}^{+}=C_{0}-\alpha_{BCA}$$

(14) $$\left[BCA\right]=C_{1}-2.\alpha_{BCA}$$

(15) $$\left[HXE\right]=C_{2}-C_{0}+\alpha_{BCA}$$

(16) $$K_{a}^{HXE}=\beta_{2}\frac{{\left[Cu\left(XE\right)\left(H_{2}O\right)\right]}^{+}}{\left[HXE\right]}\frac{{\left[BCA\right]}^{2}}{{\left[Cu{\left(BCA\right)}_{2}\right]}^{3-}}$$

(17) $$K_{a}^{HXE}=\beta_{2}\frac{\left(C_{0}-\alpha_{BCA}\right){\left(C_{1}-2.\alpha_{BCA}\right)}^{2}}{\left(C_{2}-C_{0}+\alpha_{BCA}\right)\alpha_{BCA}}$$

### 2.10 Cyclic Voltammetry Assays

A 50 mM HEPES pH 7.4 solution was used as the electrolyte solution in all experiments. Experiments were conducted under argon atmosphere, toward the reductive potential, with the initial potential of 0 mV and using 0.1 V s^−1^ as scan rate. All potentials cited in this work will be given as function of the SCE.

### 2.11 Copper Removal From Aβ

***UV‐Vis*** ─Spectra of the ternary systems HXE, Cu^2+^ and Aβ_16_/Imidazole (Im) were measured after the addition of 1.2 equivalent of HXE to a solution containing 1 equivalent of Cu^2+^ and 1.2 equivalents of Aβ_16_ peptide or 6.0 equivalents of Im. Experiments were performed in a 50 mM HEPES pH 7.4 solution. A Savitzky‐Golay smoothing was applied using a window of 50 points to reduce noise.

***EPR*** ─Samples were prepared in a 50 mM HEPES pH 7.4 solution with 10% glycerol to prevent the crystallization of the solvent and 1% DMSO to prevent precipitation in the lower temperature required for the experiment. Mixtures were made in 1500 µL falcon tubes, with a final concentration of ^65^Cu^2+^ of 500 µM. Different equivalents of HXE and/or Aβ peptide and/or Im were also added. Solutions were transferred to EPR quartz tubes and frozen in liquid nitrogen (77 K).

***NMR*** ─Samples were prepared in an Ar‐saturated glove box in phosphate buffer (200 mM, pH 7.4 in D_2_O). Aβ_16_ (500 µM in D_2_O) was first mixed with 2 eq. of Na_2_S_2_O_4_ (1 mM in D_2_O) and [Cu(CH_3_CN)_4_]BF_4_ (490 µM in CD_3_CN, 3% of the final volume) in a Eppendorf tube. **HXE** (500 µM, in D_2_O) was then added to the Eppendorf tube and 600 µL of the solution was added to an NMR tube with J Young valve.

### 2.12 Ascorbate Consumption Assay

The consumption of ascorbate (Asc) in the presence of Cu was monitored by UV‐Vis. It mirrors the production of ROS in solution, as reported previously [56, 65]. Intensity of the Asc absorption band at λ = 265 nm (ε = 14500 L mol^−1^ cm^−1^) was monitored as a function of time, with the background signal at λ = 800 nm subtracted. Experiments with different concentrations of ligand, Zn and Aβ_16_ or imidazole (Im) were performed with a final concentration in the cuvette of: [Asc] = 100 µM, [Aβ_16_] = 0 or 12 µM, [Im] = 60 µM, [Cu^2+^] = 10 µM, [Zn^2+^] = 0, 12 or 120 µM, [**HXE**] = 0, 12 or 60 µM and [HEPES] = 100 mM, pH 7.4. Cu^2+^ is added to the solution of Asc (and peptide) generating Cu^+^. The ligand is then added afterward the addition of Cu^2^⁺, when absorbance reaches nearly 1 [55, 57, 91]. The Asc consumption rate was estimated from the slope of the plot of Asc concentration (determined by the ratio between the absorbance at 265 nm and the molar absorptivity, ε = 14500 M^−^^1^ cm^−^^1^) *versus* time (in seconds), using the first 300 s following ligand addition.

### 2.13 HO^•^ Production Mediated by Copper

Coumarin‐3‐carboxylic acid (3‐CCA) was used as a probe to detect HO^•^, since the interaction of these leads to the formation of 7‐hydroxy‐coumarin‐3‐carboxylic acid (7‐OH‐CCA), which is fluorescent at 452 nm upon excitation at 395 nm. Fluorescence experiments were performed in a 96 wells plate at 25°C. The reaction was started with the addition of 20 µL of a 5 mM aqueous solution of Asc into a 50 mM phosphate buffer pH 7.4 solution containing 3‐CCA (0.5 mM), **HXE** (12 µM), Aβ_16_ (12 µM), Zn^2+^ (12 µM), and Cu^2+^ (10 µM), to a final volume of 200 µL. The fluorescence was monitored with a 3 min interval during 90 min. Four replicates of each condition were added in the plate.

### 2.14 Aggregation Kinetics of Aβ_40_

Stock solutions of CuSO_4_ and ZnSO_4_ (0.3 mM, Milli‐Q), **HXE** (0.1 mM, Milli‐Q), ThT (250 µM, Milli‐Q), and Aβ_40_ (dilution of the monomerized peptide solution with Milli‐Q water to a final concentration of 40 µM) were prepared. Self‐assembly of Aβ_40_ in HEPES buffer pH 7.4 in the absence and presence of Cu or Zn and **HXE** was followed by the fluorescence of ThT‒(*λ*_ex_ = 440 nm, *λ*_em_ = 490 nm). ThT fluorescence increase can be considered a sigmoid curve described by Equation (18), where F_0_ and *F*_max_ are the initial and maximum fluorescence intensity, respectively, and 𝑘 is the growth rate. *t*_1/2_ is defined as the time at which the fluorescence intensity reaches half of its maximum value.

(18) $$F\;\left(t\right)=F_{0}+\frac{F_{max}-F_{0}}{1+e^{-k\left(t-t_{1/2}\right)}}=F_{0}+\frac{\Delta F}{1+e^{-k\left(t-t_{1/2}\right)}}$$

The experiments were performed at 37°C and with stirring at 200 rpm (double orbital) during 15 s before each cycle (10 min) in a 384 wells plate. The reactant addition was performed in the same order as listed here to achieve a final concentration of: [HEPES] = 100 mM, [EDTA] = 20 nM, [ThT] = 10 µM, [**HXE**] = 0 or 20 µM, [Aβ_40_] = 20 µM and [Cu] = [Zn] = 0 or 18 µM. Each condition was reproduced into six different wells. To ensure the reproducibility of the impact of **HXE** in the peptide self‐assembly, the experiment was performed four times.

### 2.15 Transmission Electron Microscopy (TEM)

Samples from different conditions were collected from 384‐well plates after the ThT assays reached the fluorescence plateau (after approximately 2 days) and diluted 4 times. Formvar‐coated grids (Delta Microscopies, France) were then prepared for microscopy using a conventional negative staining method. 5 µL drop of sample was placed on the grid for 1 min, blotted and then stained with 3 µL uranyl acetate (1%) for 1 min. The grids were then mounted and examined in a TEM. The images were then processed using GATAN software.

## 3 Results and Discussion

### 3.1 Syntheses and Characterization

**FIGURE 2:**
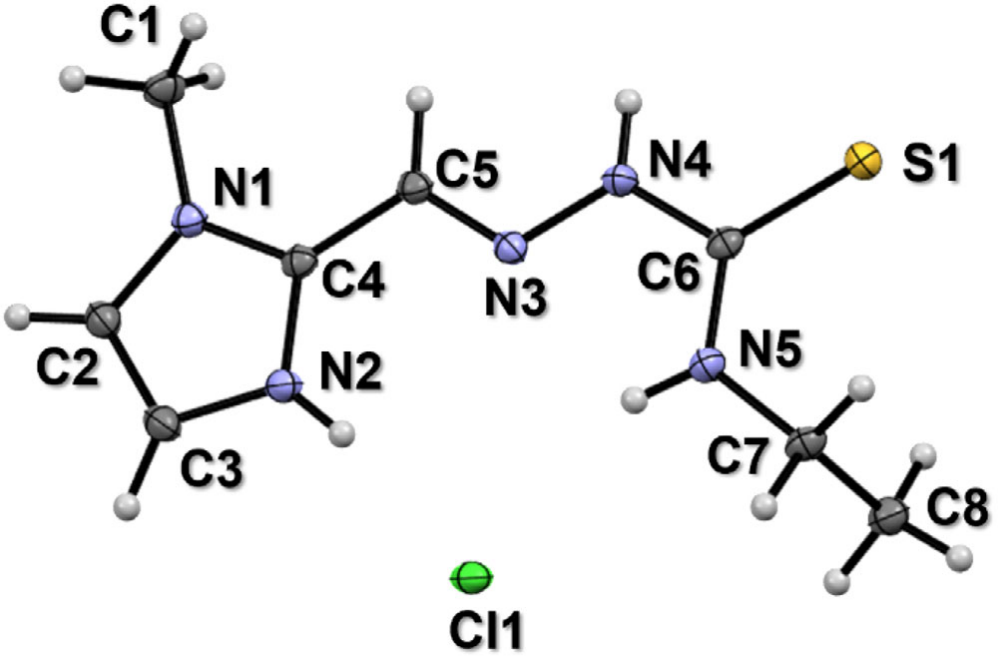
Ellipsoid plot of **HXE**, HCl. Ellipsoids drawn at 50% probability level.

**HXE** was synthetized as described in the experimental section, resulting in the precipitation of a white powder during the synthesis, corresponding to the hydrochloride form of the ligand, **HXE**, HCl. After solubilization of this powder in an 80/20 EtOH/H_2_O mixture and slow evaporation of the solvent, white needle crystals were isolated. The protonation of the imidazole nitrogen occurs due to the acid catalysis used in the synthesis (see Figure S1). **HXE**, HCl crystalizes in the monoclinic system, space group P2_1_/c and with four molecules and four chloride ions per unit cell. The structure of **HXE**, HCl is shown in Figure 2. An (*E*)‐configuration regarding the C5═N3 bond and *syn* conformation around the N4‒C6 are observed. The main crystal, data collection, and refinement parameters are given in Table S1, and some relevant bond distances and angles are reported in Table S2. The crystal network is stabilized through nonconventional hydrogen bonds involving the chloride ions, N2‒H and N5‒H from an **HXE** unit, as well as N4’‒H from a second thiosemicarbazone unit. The geometric parameters associated to such interactions are listed in Table S3. No other interaction between the protonated, cationic **HXE**, HCl units, as for example π‐π stacking, is observed (Figure S3).

**FIGURE 3:**
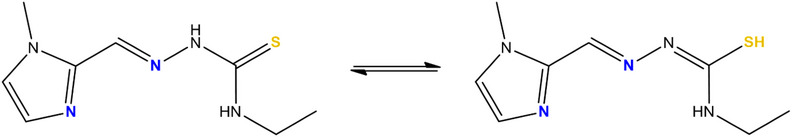
Equilibrium between the thioamide and thioiminol tautomers of **HXE**.

**FIGURE 4:**
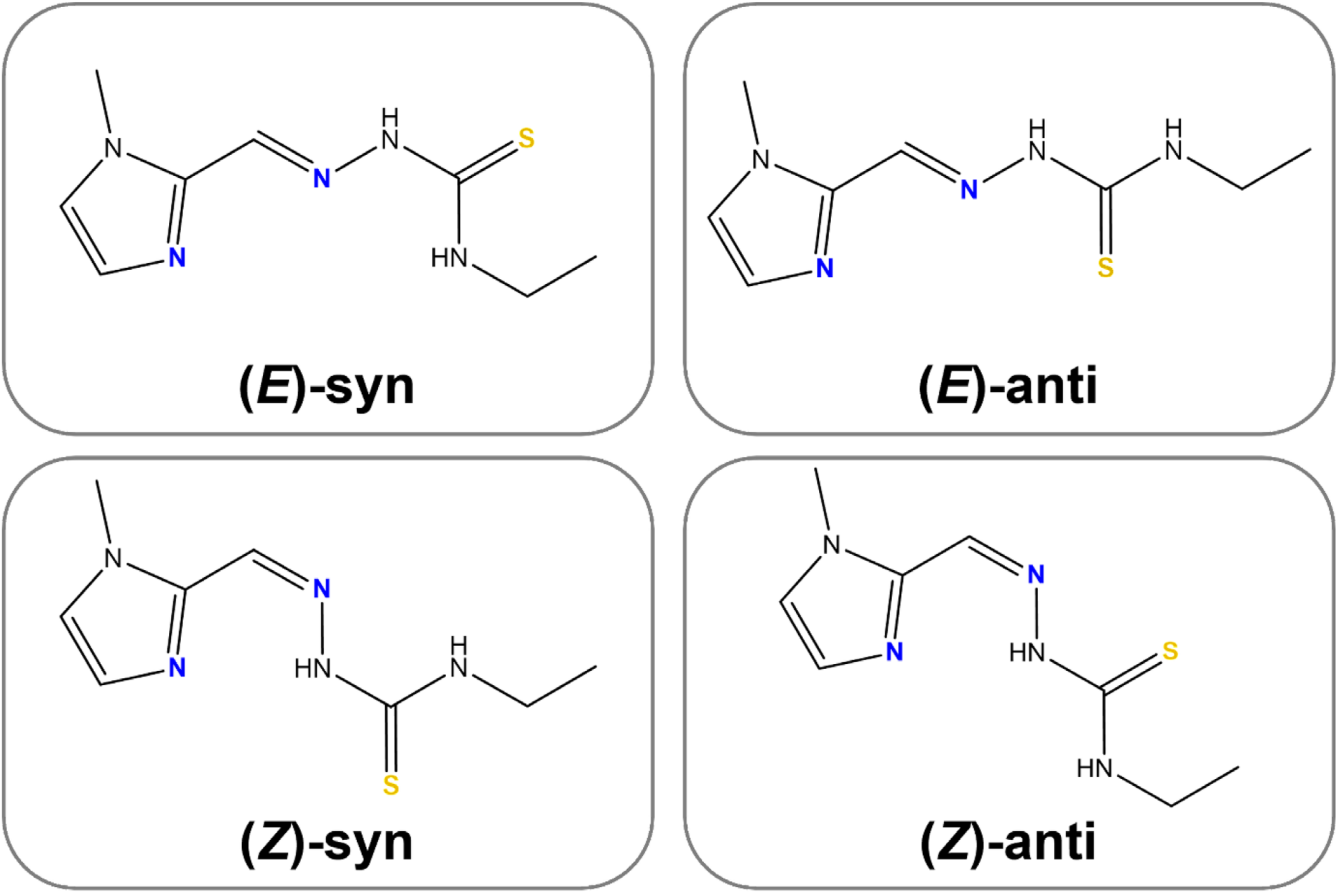
Possible (*E*)/(*Z*) isomers and syn/anti conformers of **HXE**.

In solution, **HXE** can undergo a tautomeric equilibrium between thioamido and thioiminol forms (Figure 3), which provides a partial double bond character to the C6─N4 bond, restricting free rotation around it. Consequently, in addition to the classic geometric isomerism (*E*)‐ and (*Z*)‐ around the C5═N3 bond, **HXE** can also be described in terms of *anti* and *syn* conformations, according to the relative orientation between C6─S1 and N4─H bonds, which can be pointing to opposite sides or to the same side in the plane defined by the atoms H–N4─C6─S1 (Figure 4).

To characterize the ligand in solution, ^1^H NMR in DMSO‐*d*_6_ was performed, where two sets of signals were observed, corresponding, respectively, to 97% and 3% of the total amount of **HXE** (Figure S4). The major isomer was assigned as the (*E*)‐isomer and the minor one, as the (*Z*)‐isomer, based on the chemical shifts of the N4 proton, which appears at 12.14 and 13.42 ppm, respectively. This assignment is consistent with previous reports from our research group and the literature [61, 92, 93, 94, 95]. A NOESY experiment allowed the attribution of the major species as the (*E*)‐anti isomer (Figure S5). By the ^1^H spectrum, it is also possible to conclude that the ligand was obtained with a high purity.

The stability of **HXE** in a buffered solution (50 mM HEPES pH 7.4) was followed for 24 h by UV‐Vis in order to evaluate possible hydrolysis of the C5─N3 bond. **HXE** shows a single intra‐ligand transition at 324 nm (ε = 3.17 ± 0.05 × 10^4^ M^−1^ cm^−1^), which did not show any absorbance change during the experiment (data not shown), indicating the high stability of this ligand toward hydrolysis in an aqueous buffered solution at pH 7.4. This result is in accordance with the behavior observed for other methyl‐imidazole containing ligands previously studied by our group [65, 67, 69].

**FIGURE 5:**
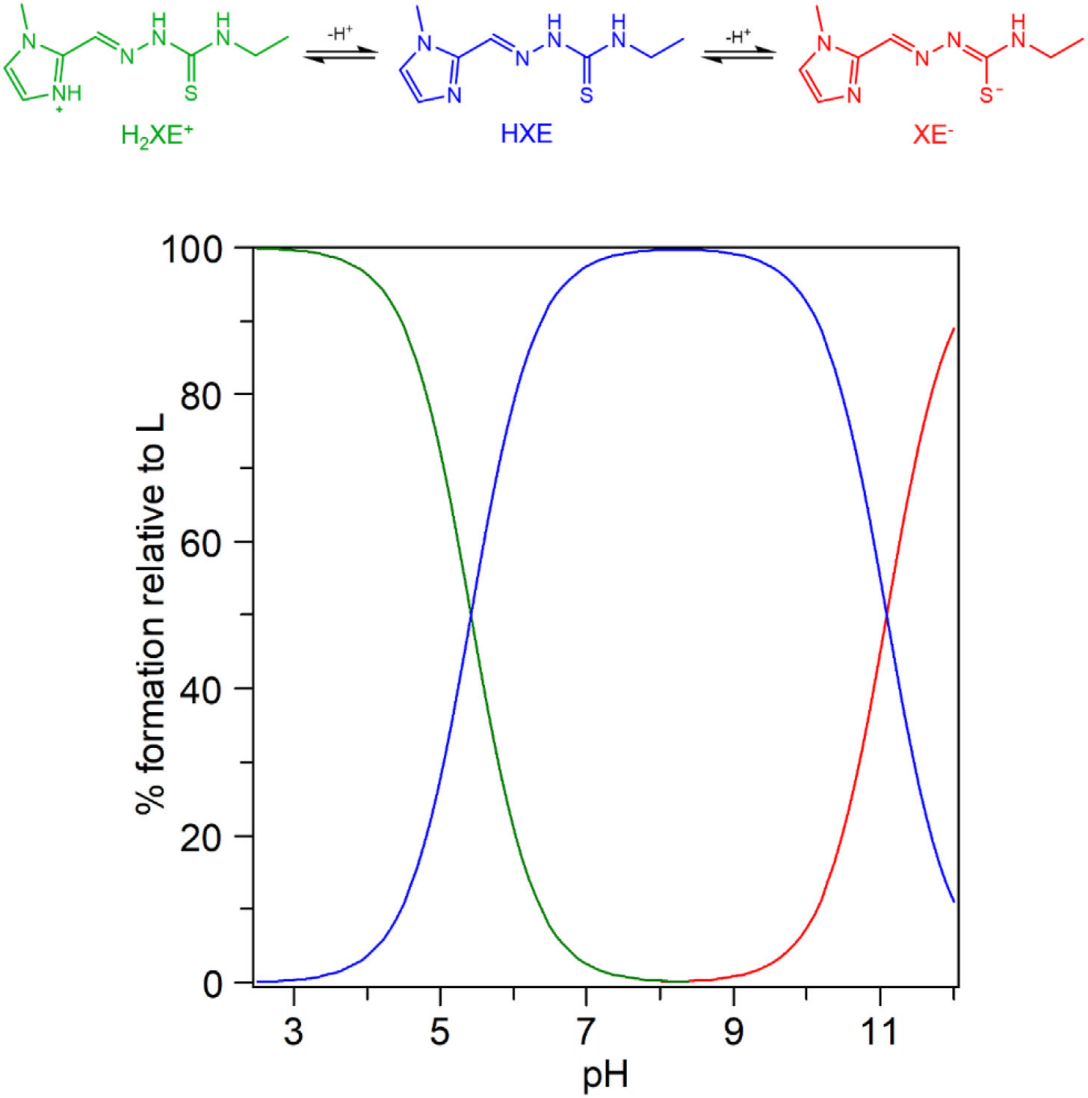
Species distribution curves of the thiosemicarbazonic ligand **HXE** as a function of pH. Experimental conditions: *I* (KCl) = 0.2 M, T = 298 K, and [**HXE**] = 2.0 mM.

Protonation constants of the ligand were determined by potentiometric titration in aqueous solution (*I* = 0.2 M, 25°C). **HXE**, HCl shows two deprotonation sites. The most acidic one can be assigned to the protonated methylimidazole group (i.e., **H_2_XE** ^+^ → **HXE** + H^+^, p*K*_a_ = 5.42), while the higher p*K*_a_ value is related to deprotonation of the thioamide group at N4 (**HXE** → **XE** ^‒^ + H^+^, p*K*_a_ = 11.09). Therefore, at physiological pH, the ligand is in its neutral **HXE** form, being this the main species between pH 6 and 11 (Figure 5).

### 3.2 Pharmacological Parameters and Toxicity

*In silico*, calculations of pharmacological parameters can be a useful tool to predict the bioavailability and permeability of new compounds. All the calculated parameters for **HXE** (Table 2) showed values in agreement with the expected range for drugs that aim to act in the CNS, according to Lipinski's rules [96].

**TABLE 2:** Pharmacological parameters calculated for **HXE** using OSIRIS Property Explorer: DataWarrior^TM^ software. MW: Molar weight, cLogP: Calculated octanol: Water partition coefficient (hydrophobicity/lipophobicity parameter), PSA: Polar surface area.

	MW (g mol^−1^)	cLog P	PSA (Å^2^)
Calculated	211.29	0.47	86.33
Expected range	200–450 [97]	0–3 [98]	< 90 [99, 100, 101]

Furthermore, **HXE** toxicity was evaluated in HT‐22 cells. In that case, exposure to up to 200 µM of ligand did not affect the viability of the hippocampal neuronal cell line, as assessed by the MTT assay [F(6, 27) = 1,284, p = 0.2976] (Figure S6).

### 3.3 Binary Systems Between HXE and Cu^+^/Cu^2+^ or Zn^2+^ Ions

The interaction between **HXE** and Cu^+^/Cu^2+^ or Zn^2+^ were investigated in the solid state for Cu^2^⁺ and in solution for the three ions, in order to assess their binding modes and affinities. In this context, [Cu(**XE**)(H_2_O)]^+^, [Cu(**XE**)(H_2_O)], and [Zn(**XE**)(H_2_O)_x_]^+^ refer to the complexes formed *in situ* by mixing a ligand solution with the respective metal ions (M = Cu^2^⁺, Cu⁺, or Zn^2^⁺) at neutral pH. Note that for Cu^+^, we employ the [Cu(**XE**)(H_2_O)] notation for the sake of simplicity, but there is no data about its symmetry or showing that Cu^+^ induce the deprotonation of **XE**, in contrast to Cu^2+^ and Zn^2+^ (see below).

**FIGURE 6:**
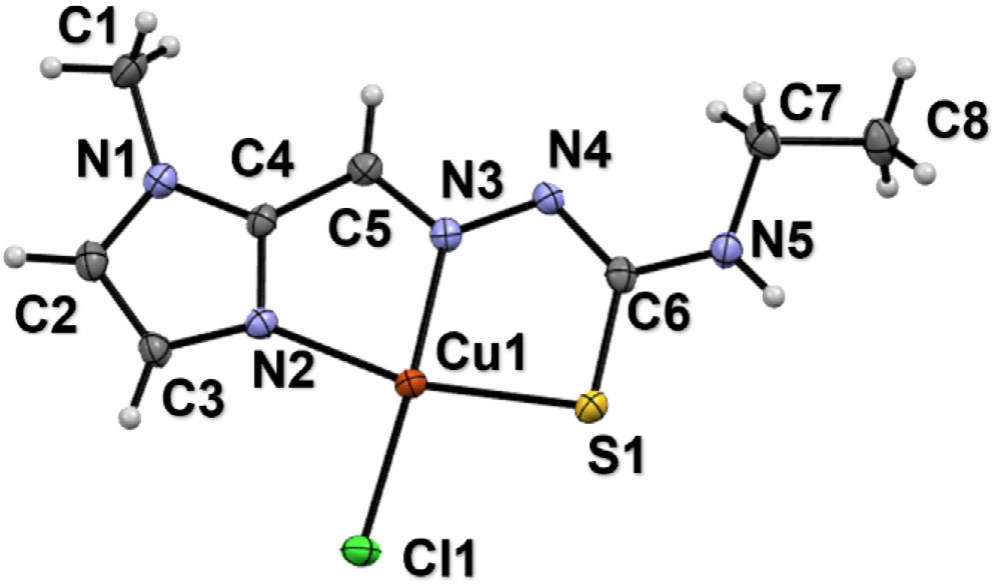
Ellipsoid plot of [Cu(**XE**)Cl]. Ellipsoids drawn at 50% probability level.

We were able to isolate a monocrystal of the 1:1 complex, [Cu(**XE**)Cl], allowing the complete characterization of its structure in the solid state. The main crystal, data collection, and refinement parameters for [Cu(**XE**)Cl] are given in Table S4. The complex adopts a square‐planar geometry (τ_4_ = 0.14) [102], in which the fully deprotonated form of the (*E*)‐anti ligand binds to Cu^2+^ *via* the methylimidazole and thiosemicarbazone moieties (N2, N3, and S1). This indicates that Cu^2+^ binding induces deprotonation of **HXE**, which is coordinated in its thioiminolate form. The coordination sphere of Cu^2+^ is completed by a chloride ion (Figure 6), resulting in the neutral complex [Cu(**XE**)Cl]. It is worth noting that the binding of the chloro ligand is expected not to be kept in aqueous solution, where a water ligand will replace it in line with much higher concentration. Bond distances and angles around the metal center can be seen in Table S5. The decrease in the N4─C6 bond length and increase of C6‒S1 is in full accordance with electron delocalization due to deprotonation of N4 (thioiminolate coordination).

**FIGURE 7:**
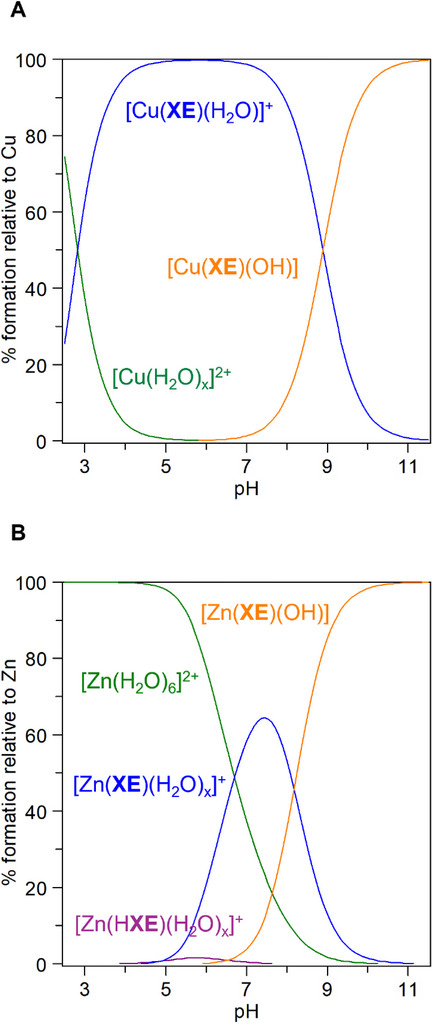
Species distribution curves of complexes formed in (**A**) 1:1 Cu^2+^:**HXE** and (**B**) 1:1 Zn^2+^:**HXE** system as a function of pH. Simulation conditions: [**HXE**] = 50 µM, [Cu^2+^] = [Zn^2+^] = 50 µM.

The formation of **HXE**‐based complexes in the presence of equimolar amounts of Cu^2+^ and Zn^2+^ ions was further studied in aqueous solution by potentiometry. The stability constants calculated are listed in Table 3, in which the protonation constants of the ligand were included for comparison. The best fits (Figure S7) for the titration data were obtained for complex species ML^+^ and MLH_‒1_ for Cu^2+^ and MLH^2+^, ML^+^, and MLH_‒1_ for Zn^2+^. Simulated species distribution curves for a metal:ligand ratio of 1:1 are shown in Figure 7.

**TABLE 3:** Protonation constants of the ligand and stability constants for its Cu^2+^ and Zn^2+^ complexes (standard deviations in parentheses). Experimental conditions: *I* (KCl) = 0.2 M, T = 298 K, and [**HXE**] = 2.0 mM. In the case of the stability constants, [Cu^2+^] or [Zn^2+^] = 2.0 mM.

	log β	
Species	**Cu^2+^**	**Zn^2+^**
H_2_L^+^	16.51(10)	
HL	11.09(7)	
MLH^2+^	‒	13.89(15)
ML^+^	15.47(5)	9.02(3)
MLH_‐1_	6.58(8)	0.84(5)

For Cu^2+^, the species ML^+^, [Cu(**XE**)(H_2_O)]^+^, in which the ligand is already fully deprotonated, starts to be formed below pH 3, dominates between pH 3 and ∼9, and lasts until pH 10 (Figure 4A). This is, by far (>95%), the main complex present at physiological pH. Deprotonation of the equatorial coordinated water, leading to the neutral hydroxo complex MLH_‒1_, [Cu(**XE**)(OH)], occurs at a higher pH: p*K*_a [Cu(_ **_XE_**_)(H2O)]_^+^_/[Cu(_ **_XE_**_)(OH)]_ = 8.89. It is worth noting that the CuL species formed from **HXE** is 3 orders of magnitude more stable (15.47 *vs*. 12.49) than the corresponding one formed by the similar, tridentate (N_2_O‐donor) 1‐methylimidazole‐containing *N*‐acylhidrazonic ligand HX1Diox [65], stressing the importance of the sulfur atom in the thiosemicarbazone **HXE** (which is a N_2_S‐donor) for the stabilization of the Cu^2+^ complex.

Different from Cu^2+^, the formation of Zn^2+^ complexes only starts at pH around 5 (Figure 4B). In more acidic solutions, the aquo complex [Zn(H_2_O)_6_]^2+^ is virtually the unique Zn^2+^ existing form. MLH^2+^ is only marginally present. ML^+^ species [Zn(**XE**)(H_2_O)_x_]^+^, on the other hand, dominates between pH 6.7 and 8.2, peaks at pH 7.5 (65%) and lasts until pH 9.5. By increasing the pH, deprotonation of a coordinated water molecule also occurs near pH 8.2, leading to the formation of the hydroxo complex [Zn(**XE**)(OH)], in which coordinated water molecules were excluded for the sake of simplicity, starting at around pH 7. This species becomes predominant from pH 8.2 on. It is interesting to observe that the deprotonation of [Zn(**XE**)(H_2_O)_x_]^+^ occurs at a lower pH than that of [Cu(**XE**)(H_2_O)]^+^, which would not be expected based on the HSAB principle. However, although Cu^2+^ is usually considered a slightly stronger Lewis's acid than Zn^2+^, this property is dependent on the coordination geometry and also on the nature of ligands. Mareque‐Rivas *et al.*, for example, reported p*K*_a_ values ranging from 7.74 to 5.99 for coordinated water in a series of zinc(II) complexes containing ligands derived from tpa [tris(pyridylmethyl)amine] [103]. Moreover, Zn^2+^ forms more stable complexes with softer donors (as **HXE**) than with the harder ones, and this may affect the ability of coordinated water to undergo deprotonation.

**FIGURE 8:**
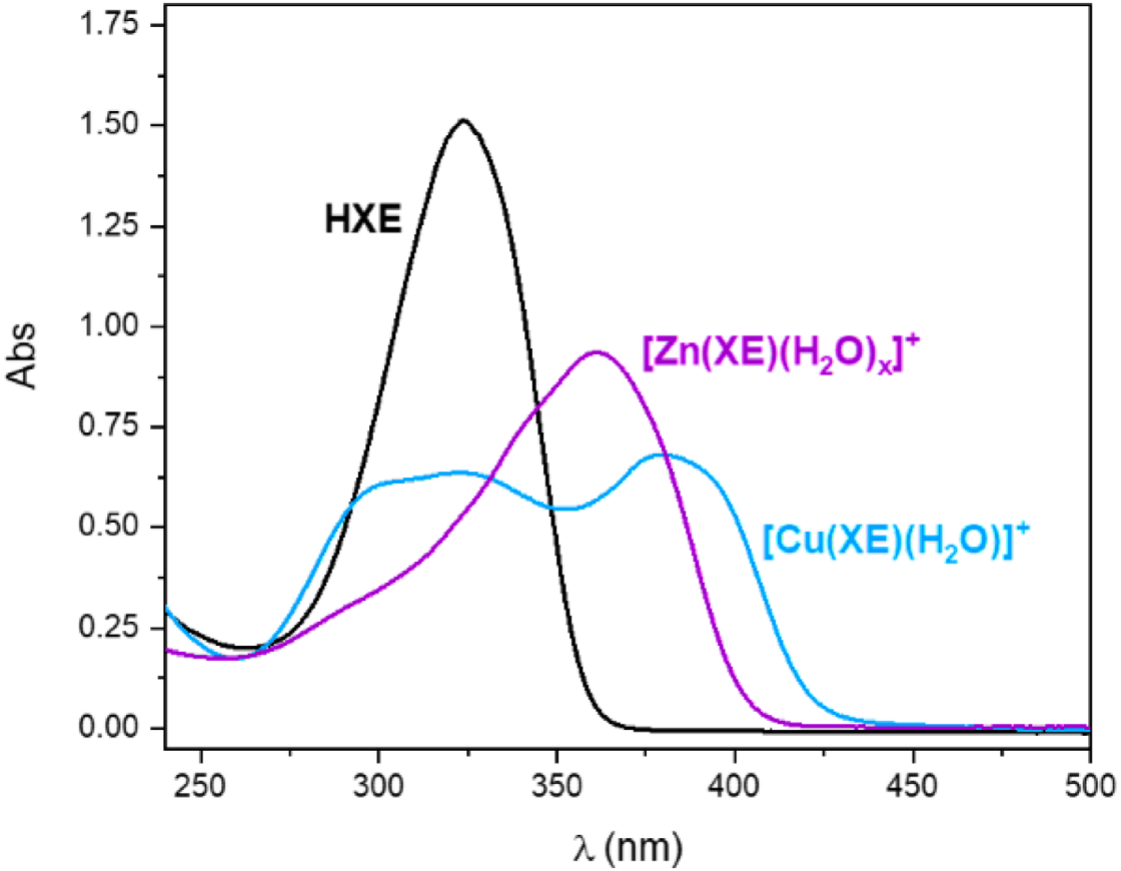
UV‐Vis spectra of **HXE** and its Cu^2+^ and Zn^2+^ complexes. [**HXE**] = [Cu^2+^] = 50 µM, [Zn^2+^] = 250 µM in 50 mM HEPES pH 7.4.

The spectroscopic profile of the ligand and its Cu^2+^ and Zn^2+^ complexes formed *in situ* at pH 7.4 were investigated through UV‐Vis (Figure 8). Upon coordination with one equivalent of either Cu^2+^ or Zn^2+^, the intra‐ligand band of **HXE** at 324 nm decreases in intensity, and a new LMCT band emerges at 383 nm and 362 nm, respectively. The maximum absorption wavelengths, molar absorptivities, and the corresponding transition assignments for **HXE** and its Cu^2^⁺ and Zn^2^⁺ complexes are summarized in Table 4.

**TABLE 4:** UV‐Vis parameters for complexes [Cu(**XE**)(H_2_O)]^+^ and [Zn(**XE**)(H_2_O)_x_]^+^ in 50 mM HEPES pH 7.4 solution.

	*λ*_max_ (nm)	ε (M^−1^ cm^−1^)	Transition
HXE	324	31700	Intra‐ligand
[Cu(XE)(H_2_O)]^+^	383 626	13680 164	LMCT *d*‐*d*
[Zn(XE)(H_2_O)_x_]^+^	362	19690	LMCT

**FIGURE 9:**
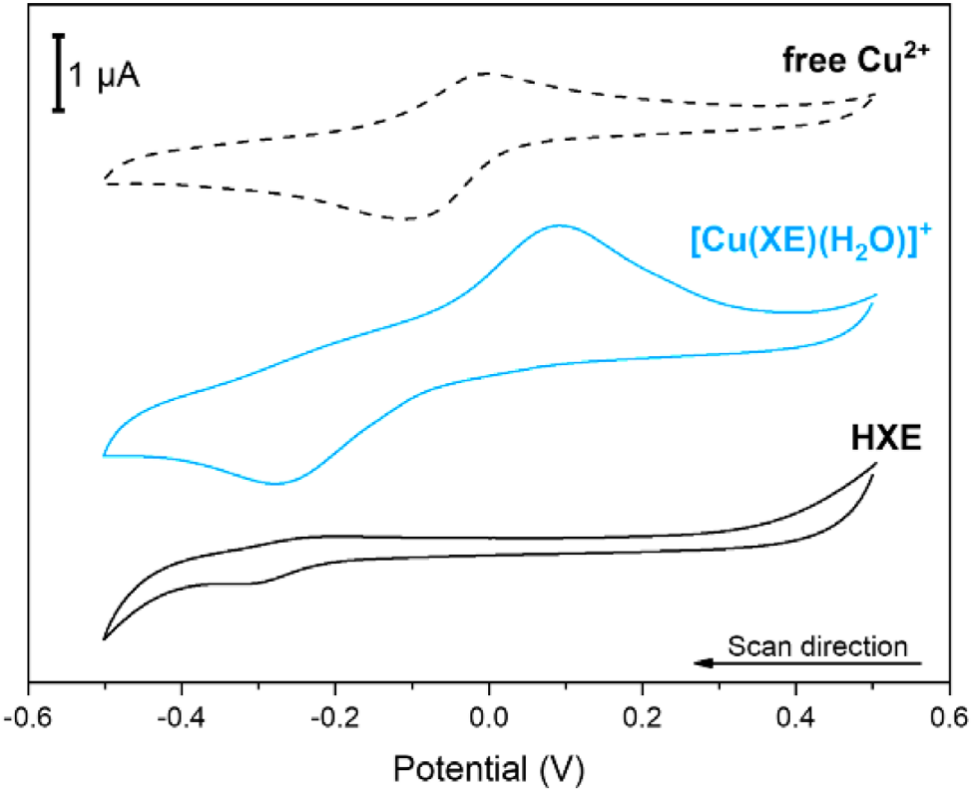
(**A**) Voltammograms of free Cu^2+^ (black dotted line), [Cu(**XE**)(H_2_O)]^+^ (light blue line) and **HXE** (black line) in 50 mM HEPES pH 7.4 with a scan rate of 100 mV s^−1^. Electrodes: glassy carbon (WE) platinum wire (CE), and saturated calomel electrode (RE).

**HXE** and [Cu(**XE**)(H_2_O)]^+^ were also electrochemically evaluated in a buffered solution pH 7.4 (Figure 9). **HXE** shows a weak reduction process around ‐250 mV (*vs*. SCE). With the addition of Cu^2+^, a process displaying *E*_pa_ = +90 mV and *E*_pc_ = ‒270 mV is observed, in which peak‐to‐peak separation indicates electrochemical irreversibility. When compared with “free” Cu^2+^ in buffer, an increase in ∆*E* due to the coordination of **HXE** is observed, going from approximately 93 in “free copper” to 360 mV in the complex, showing that the ligand makes the redox cycle between Cu^2+/+^ sluggish. The increase in the scan rate (Figure S8A) causes an increase in the separation between the anodic and cathodic peaks (Δ*E*), which depends linearly on the square root of the scan rate (Figure S8B), indicating a diffusion controlled process [104]. The irreversibility of the process may originate from different geometries between Cu^2+^ and Cu^+^ bound to **HXE**.

**FIGURE 10:**
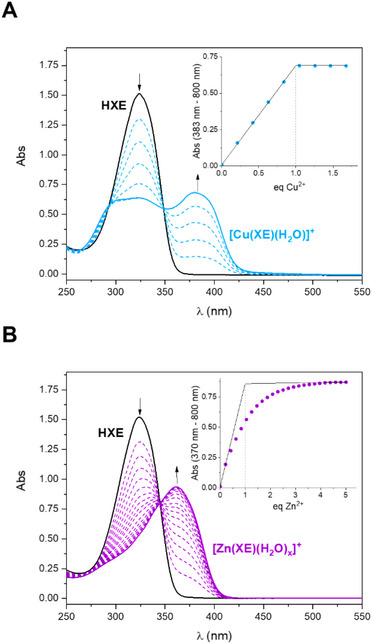
UV‐Vis spectra of **HXE** solution after the addition of increasing amounts of (**A**) Cu^2+^ and (**B**) Zn^2+^. [**HXE**] = 50 µM in 50 mM HEPES pH 7.4.

UV‐Vis titrations of Cu^2+^ and Zn^2+^ over a **HXE** solution were performed in order to confirm the stoichiometry and apparent affinity of the complex formed *in situ* at physiological pH (log *K*_app_) and deduce the selectivity of the ligand. The electronic spectra for both metal ions are shown in Figure 10. The complexation of the metal ions can be followed by the appearance of the LMCT band centered at 383 nm (ε = 13680 M^−1^ cm^−1^) for Cu^2+^ and 362 nm (ε = 19690 M^−1^ cm^−1^) for Zn^2+^. For both metal ions, the formation of 1:1 complexes under these conditions is observed (see inset in Figure 10). For Cu^2+^, the concentration range used for the experiments does not allow to have an equilibrated reaction and, thus, the affinity cannot be determined. In contrast, the apparent affinity for Zn^2+^ could be evaluated as described in the methodology section (Figure S9), obtaining log *K*_app_ {[Zn(**XE**)(H_2_O)_x_]^+^} = 5.0 ± 0.1.

**FIGURE 11:**
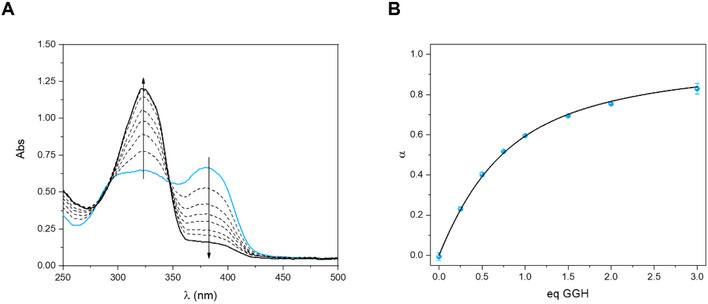
(**A**) Selected UV‐Vis spectra of a solution of [Cu(**XE**)(H_2_O)]^+^ with the addition of different equivalents of GGH. (**B**) Progression of Cu(GGH) formation as a function of the number of equivalents of competitor and theoretical curve (best fit). Mean and standard variation between the replicates are shown. [**HXE**] = 50 µM, [Cu^2+^] = 45 µM in 50 mM HEPES pH 7.4.

To determine the conditional affinity of **HXE** for Cu^2+^ ions at pH 7.4, competition assay was performed using the well‐known ATCUN (Amino‐Terminal Cu and Ni binding motif) peptide GGH [105, 106, 107]. This peptide binds Cu^2+^ with a square planar geometry through four nitrogen atoms (the terminal amine, the N^δ^ of the Im ring of histidine at the third position and the two deprotonated amides in peptide backbone in between) [107, 108]. A value of 12.7 for log *K*_GGH‐Cu_ was determined by Noormägi *et al.* using competition with HSA and monitored by LC‐ICP‐MS [109]. This value is consistent with the binding constant previously determined by a thorough potentiometric study, showing a value of 12.215 ± 0.001 at pH 7.4 but for the nonamidated GGH peptide [110]. Our experimental design involved the addition of different GGH equivalents to a buffered [Cu(**XE**)(H_2_O)]^+^ solution (pH 7.4). Progression of the reaction, that is [Cu(GGH)]/C_0_, was determined by the disappearance of the absorbance at 383 nm relative to [Cu(**XE**)(H_2_O)]^+^ complex. Equilibrium was reached after 180 min (Figure S10). The spectra resulting of successive additions of competitor are shown in Figure 11. Progression of the reaction for each mixture was then plotted in function of the number of equivalents of peptide and the experimental data was fitted using the respective quadratic equation to determine the stability constant of [Cu(**XE**)(H_2_O)]^+^, obtaining log *K*_cond_ = 12.3 ± 0.1.

For both Cu^2+^ and Zn^2+^, the apparent and conditional affinity constants determined by direct titration and competition experiments, respectively, are in agreement with those deduced from the potentiometric titration (11.8 and 5.4, respectively, at pH 7.4).

To estimate the affinity of **HXE** toward Cu^+^, competition with the well‐known BCA ligand was performed. BCA forms an ML_2_ complex with Cu^+^, with an affinity of log β_2_ = 14.7 or 17.2 [111, 112]. With the addition of **HXE**, a decrease in the characteristic absorption at 562 nm, related to [Cu(BCA)_2_]^3‒^, is observed (Figure S11). Absorbance at this wavelength was used to calculate the percentual of Cu^+^ that remains bound to BCA. The apparent stability of [Cu(**XE**)(H_2_O)] could be determined as described in Equation (17), resulting in a value of log *K*_app_ = 8.7 ± 0.1 or 11.2 ± 0.1, based on either affinity values reported for the BCA complex.

All affinity constants determined for **HXE** in this work and the ones found in the literature for Aβ are summarized in Table 5. Since the values reported in literature for the BCA competitor differ (log β_2_ = 14.7 or 17.2), the resulting affinities of **HXE** or Aβ change accordingly. Hence, to directly compare the Cu^+^ affinity for **HXE** and Aβ, either reference value (14.7 or 17.2) was used. This leads to the same difference of Cu^+^ affinity between them, *id est*, between one and two orders of magnitude (8.7 *vs*. 6.9, and 11.2 *vs*. 10). On the other hand, **HXE** has an affinity for Cu^2+^ approximately 100–1000 times greater than that of Aβ. With respect to selectivity [s = *K*_app_ (M)/*K*_app_ (Zn^2+^), where M = Cu^2+^ or Cu^+^], the Cu^2+^ *versus* Zn^2+^ selectivity of **HXE** is about 1000 times higher and the Cu^+^ *versus* Zn^2+^ selectivity is about 100 times higher compared to the peptide.

**TABLE 5:** Copper and zinc binding affinities with **HXE** and Aβ peptide.

	HXE		Aβ
Metal ion	Log *K*_app_	Experimental conditions	Log *K*_app_
Cu^2+^	12.3 ± 0.1	by competition with the GGH peptide (50 mM HEPES pH 7.4)	9 – 10 [43, 44, 45, 46]
	11.8	by potentiometric titration (at pH 7.4)	
Cu^+^	8.7 ± 0.1	by competition with BCA (50 mM HEPES pH 7.4 0.3% ACN)	6.9 [47]
	11.2 ± 0.1		∼10 [46, 48, 49]
Zn^2+^	5.0 ± 0.1	by spectrophotometric titration (50 mM HEPES pH 7.4)	5 – 6 [113, 114, 115]
	5.4	by potentiometric titration (at pH 7.4)	

Based on the evaluated affinity values, the **HXE** ligand is expected to remove Cu^+^ and Cu^2+^, but not Zn^2+^ from the corresponding Aβ complexes. It is worth noting that, for both oxidative states of copper, **HXE** is not expected to effectively compete for the metal ion against their main biological transporters: HSA, in the case of Cu^2^⁺ (*K*_d_ ∼ 10^−13^ M) [110], and Ctr1, in the case of Cu^+^ (*K*_d_ ∼ 10^−14^ M) [116].

### 3.4 Copper Removal From Aβ_16_ by HXE

**FIGURE 12:**
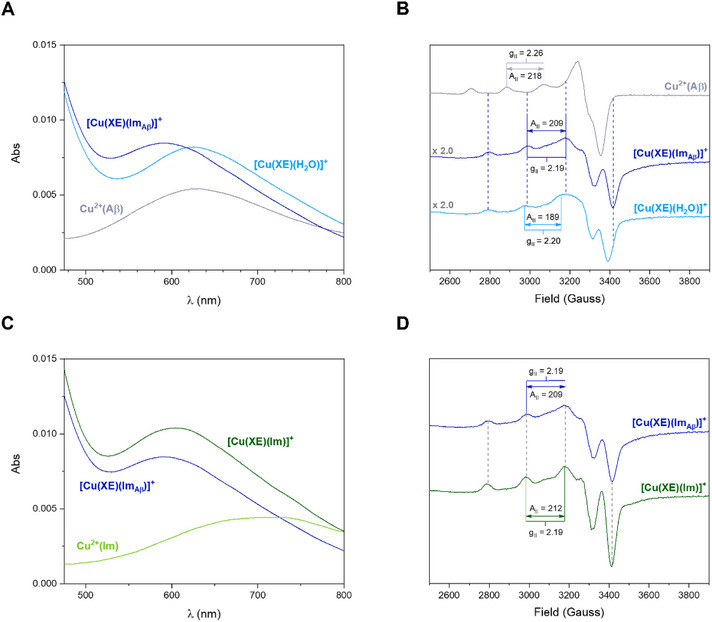
Comparison of UV‐Vis and low‐temperature (120 K) X‐band EPR spectra of [Cu(**XE**)(Im_Aβ_)]^+^ with the binary systems (**A** and **B**, respectively) and with the ternary system [Cu(**XE**)(Im)]^+^ (**C** and **D**) at pH 7.4.UV‐Vis: [**HXE**] = [Aβ] = 60 µM, [Im] = 300 µM, [Cu^2+^] = 50 µM in 50 mM HEPES pH 7.4. EPR: [**HXE**] = [Aβ] = 600 µM, [Im] = 3000 µM, [^65^Cu] = 500 µM in 50 mM HEPES pH 7.4 1% DMSO 10% glycerol.

Once the affinity of **HXE** for metal ions was evaluated, the ability of the ligand to abstract the metal ion from Aβ peptide was directly studied by UV‐Vis and EPR for Cu^2+^ (Figure 12A, 12B). By UV‐Vis, both Cu^2+^(Aβ_16_) and [Cu(**XE**)(H_2_O)]^+^ show a *d*‐*d* band centered at ∼630 nm. The addition of **HXE** to a Cu^2+^(Aβ) solution results in a shift of this band to ∼595 nm, which could suggest the formation of a ternary complex. EPR spectra of **HXE** added to Cu^2+^(Aβ_16_) shows a different profile when compared to the complexes of reference, with the total disappearance of Cu^2+^(Aβ_16_) signatures and the appearance of a new signature close to those observed for the [Cu(**XE**)(H_2_O)]^+^ species, but meaningfully different. Hence, the formation of the ternary species [Cu(**XE**)(Im_Aβ_)]^+^ is inferred. To confirm the formation of a ternary complex in which the equatorial coordination of [Cu(**XE**)(H_2_O)]^+^ is completed by an Im ring from one of the His residues of Aβ, further experiments were performed using Im in the place of the peptide. In that case, through both techniques, similar behaviors were observed (Figure 12C, 12D), confirming the formation of a ternary complex under the experimental conditions used. The spectroscopy parameters for all the species mentioned above are summarized in Table 6. Proposed structure for [Cu(**XE**)(Im_Aβ_)]^+^ is shown in Figure S12.

**TABLE 6:** UV‐Vis and EPR parameters obtained for the binary systems Cu^2+^(Aβ_16_) and [Cu(**XE**)(H_2_O)]^+^ and the ternary systems [Cu(**XE**)(Im_Aβ16_)]^+^ and [Cu(**XE**)(Im)]^+^, in a 50 mM HEPES pH 7.4 1% DMSO 10% glycerol solution.

	UV‐Vis			EPR	
	*λ*_max_ (nm)	ε (M^−1^ cm^−1^)	Transition	g_II_	A_II_ (10^−4^ cm^−1^)
[Cu(XE)(H_2_O)]^+^	383 626	13680 164	LMCT *d*‐*d*	2.20	189
[Cu(XE)(Im)]^+^	604	208	*d*‐*d*	2.19	212
[Cu(XE)(Im_Aβ16_)]^+^	591	169	*d*‐*d*	2.19	209
Cu^2+^(Aβ_16_)	633	108	*d*‐*d*	2.26	218

**FIGURE 13:**
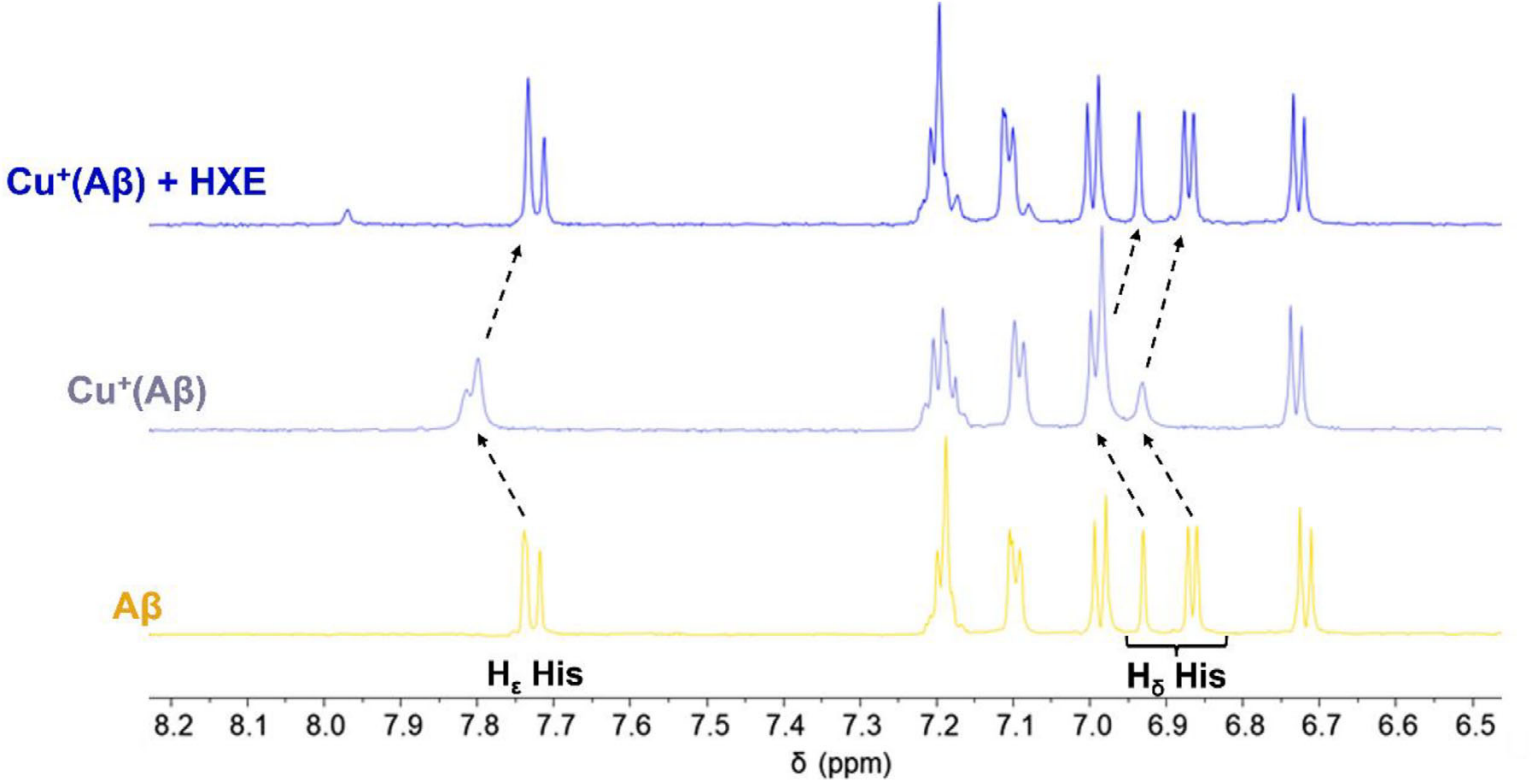
^1^H NMR spectra of Aβ_16_ (yellow), Cu^+^(Aβ_16_) (grey) and Cu^+^(Aβ_16_) + **HXE** (blue).

Competition for Cu⁺ between Aβ_16_ and **HXE** was monitored by ^1^H NMR (Figure 13). As previously described [28, 56, 117, 118], Cu⁺ coordination to Aβ_16_ induces a clear de‐shielding of the histidine proton signals involved in metal binding (observed at ∼ 6.85, 6.92, and 7.72 ppm for the apo‐peptide). However, upon addition of 1 equivalent of **HXE**, the position of the signals corresponding to the apo‐Aβ_16_ are fully restored.

[Aβ_16_] = [**HXE**] = 500 µM, [Cu^+^] = 490 µM in 200 mM phosphate buffer pH 7.4 97% D_2_O 3% ACN‐*d*_3_ in the presence of 2 equivalents of sodium dithionite.

Solutions were prepared anaerobically in an argon purged glove box.

These results highlight **HXE** efficiency in preventing Cu(Aβ) interactions, fully removing Cu^+^ from the peptide and, instead, forming a ternary [Cu(**XE**)(Im_Aβ_)]^+^ complex with Cu^2+^, indicating a potential ability to mitigate Cu‐induced Aβ toxicity.

### 3.5 ROS Production in the Presence of HXE

**FIGURE 14:**
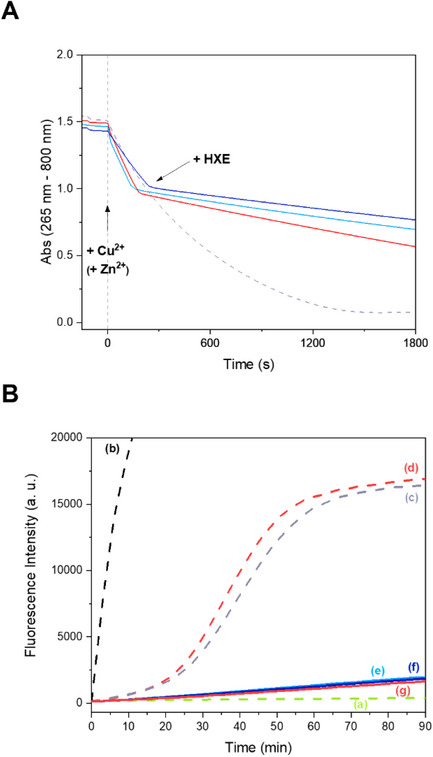
(**A**) Ascorbate consumption for: Cu(Aβ_16_) (grey dotted curve), [Cu(**XE**)(H_2_O)]^+^ (light blue curve), Cu(Aβ_16_) + 1 equivalent **HXE** (dark blue curve) and Cu(Aβ_16_) + 10 equivalents Zn^2+^ + 1 equivalent **HXE** (red curve). Ascorbate is first added, then the Aβ peptide, and the metal ions. **HXE** is added when the absorbance reaches about 1.0. [**HXE**] = [Aβ_16_] = 12 µM, [Cu^2+^] = 10 µM, [Zn^2+^] = 120 µM, [Asc] = 100 µM in 100 mM HEPES pH 7.4. (**B**) Fluorescence curve of the formation of 7‐OH‐CCA induced by Cu^2+^. (a) Control – 3‐CCA + Asc, (b) Cu, (c) Cu(Aβ_16_), (d) Cu(Aβ_16_) + Zn^2+^, (e) [Cu(**XE**)(H_2_O)]^+^, (f) Cu(Aβ_16_) + **HXE** and (g) Cu(Aβ_16_) + Zn^2+^ + **HXE**. [**HXE**] = [Aβ_16_] = [Zn^2+^] = 12 µM, [Cu^2+^] = 10 µM, [Asc] = 500 µM, [3‐CCA] = 500 µM in 50 mM phosphate buffer pH 7.4.

Oxidation of Asc by Cu^2+^ or Cu(Aβ_16_) leads to the formation of Cu^+^ in solution, which, in aerobic conditions, acts as a catalyst in the formation of ROS through the stepwise and incomplete reduction of dioxygen. Thus, the loss of the characteristic Asc absorption at 265 nm has been widely used to mirror the production of ROS mediated by Cu^2+^ or Cu(Aβ_16_) and to assess the ability of ligands to prevent this process [28, 46, 55, 56, 60, 65, 67, 91, 119, 120, 121, 122]. In the presence of Aβ and copper, the total consumption of Asc (initially at 100 µM) occurs after approximately 1500 s after the addition of the metal ion (Figure 14A—grey dotted curve) [56]. Herein, **HXE** was added to an ongoing Asc consumption reaction, when the absorbance was equal to 1. With the addition of 1 equivalent of **HXE** into this system, a significant decrease in the rate of Asc consumption is observed, resulting in a 10‐fold slower ROS production. (Figure 14A—dark blue curve).

It is worth noting that, in the experiment performed in the absence of Aβ (Figure 14A—light blue curve), the same rate of Asc consumption is observed, in line with the previously observed complete Cu^+^ removal form Aβ. In addition, this rate is much lower than that of “free copper” (about 20x faster than for the complex [Cu(**XE**)(H_2_O)]^+^). This agrees with a much more sluggish redox process of copper when bound to **HXE**, as previously detected by cyclic voltammetry. In the case of Cu^2+^, that is present at some extent in the medium, the rate of Asc consumption was similar in the absence or presence of Aβ, indicating that the ternary species [Cu(**XE**)(Im_Aβ_)]^+^ characterized by UV‐Vis and EPR has the same ability to consume Asc than [Cu(**XE**)(H_2_O)]^+^ and/or that the concentration of the ternary species in the Asc consumption experiments is too low to have an impact in the ROS generation process. Based on the fact that the ternary species was still detected by UV‐Vis at 20 µM concentration (Figure S13), the first hypothesis is preferred.

The experiment was also performed in the presence of 10 equivalents of zinc ions as a competitor for the ligand (Figure 14A—red curve). In this case, an increase in the rate of Asc consumption is observed, nearly twice as fast as in the absence of Zn^2^⁺, although still significantly slower than the control containing only Cu(Aβ_16_).

Furthermore, the generation of hydroxyl radicals (HO^•^) mediated by copper was indirectly monitored by the formation of the fluorescent product 7‐OH‐CCA over time in the presence of the metal ion, 3‐CCA and Asc (Figure 14B). In this case, in the presence of 1 equivalent of **HXE**, a significant decrease in HO^•^ production was observed and neither Aβ nor Zn^2^⁺ affected the **HXE** ability to lessen HO^•^ formation. These results are entirely consistent with those obtained in the Asc assay, demonstrating that **HXE** effectively reduces Cu(Aβ_16_)‐induced ROS generation.

### 3.6 Impact of HXE on the Aβ_40_ Aggregation Modulated by Cu^2+^ and Zn^2+^

**FIGURE 15:**
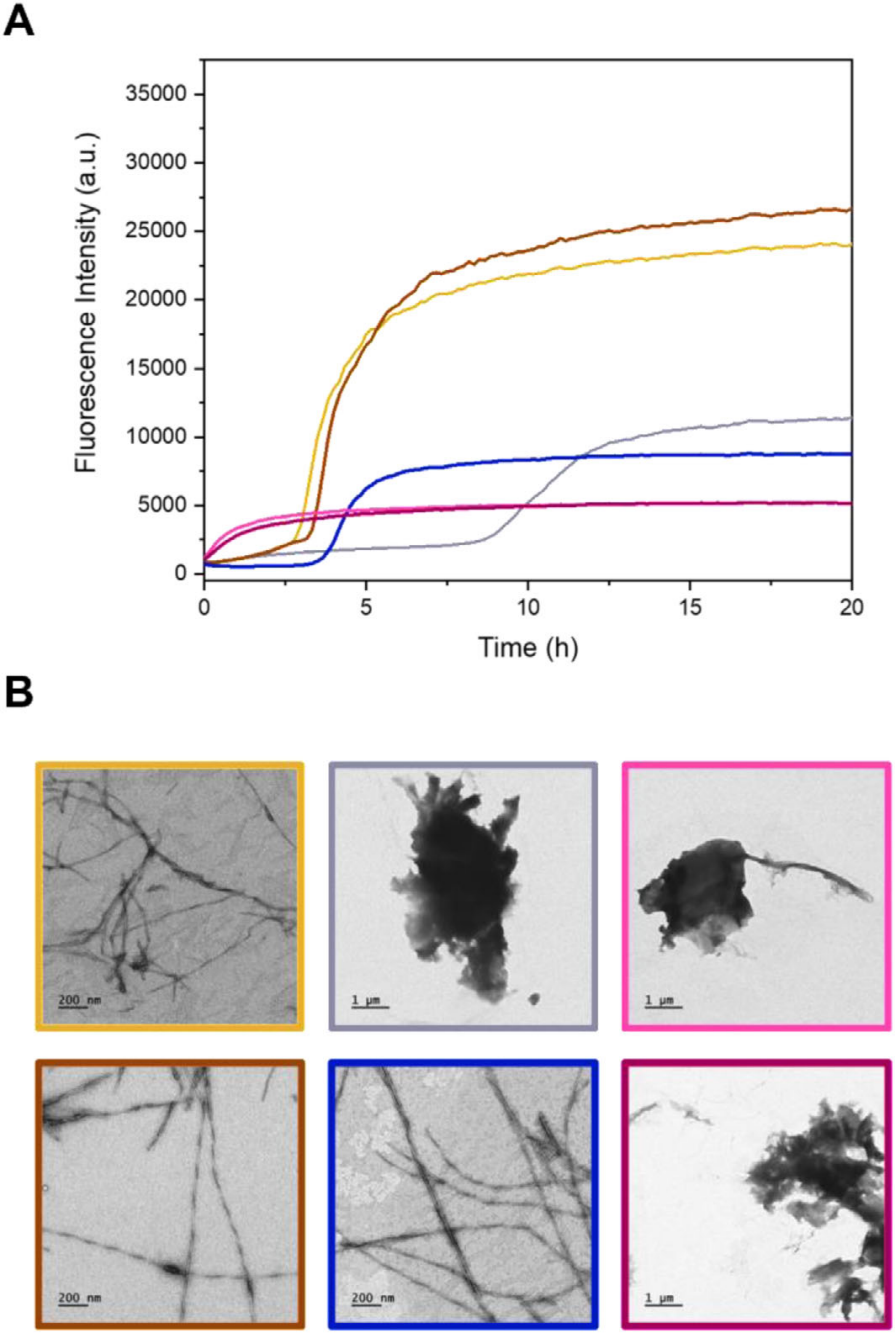
(**A**) Average of aggregation kinetic curves and (**B**) corresponding TEM images of Aβ_40_ (yellow), Cu^2+^(Aβ_40_) (grey), Zn^2+^(Aβ_40_) (violet), Aβ_40_ + **HXE** (brown), Cu^2+^(Aβ_40_) + **HXE** (blue) and Zn^2+^(Aβ_40_) + **HXE** (pink) in 100 mM HEPES pH 7.4. [Aβ] = [**HXE**] = 20 µM, [Cu^2+^/Zn^2+^] = 18 µM, [ThT] = 10 µM and [EDTA] = 20 nM.

Finally, the effect of **HXE** on the aggregation of Aβ_40_ was evaluated in the absence and presence of Cu^2+^ and Zn^2+^ using ThT as the classical fluorescent reporter, that allows to monitor the kinetics of self‐assembly of the peptide (Figure 15A) [123, 124, 125, 126, 127], and through TEM [67, 128, 129, 130, 131], in order to characterize the morphology of the aggregates (Figure 15B). For the apo‐peptide, a sigmoidal ThT curve is obtained, along with the formation of long and twisted fibrils as observed by TEM. With the addition of **HXE** on Aβ_40_, no difference in the aggregation kinetics or in the microscopy image is observed, which leads to the hypothesis that the ligand does not interact with the apo‐peptide. The kinetics of Cu^2+^(Aβ_40_) displays a different profile. As already observed in other studies, the presence of 0.9 equivalent of Cu^2+^ promoted a two‐step process with a first rapid and weak ThT fluorescence increase and a second sigmoidal process that occurs later compared to the apo‐Aβ [132, 133, 134]. The decrease in the fluoresce intensity were related to the stabilization of more amorphous aggregates, as also observed in TEM images [23, 24, 26, 135, 136]. The addition of 1 equivalent of **HXE** on the Cu^2+^(Aβ_40_) system leads to a sigmoidal curve with a similar t_1/2_ as apo‐Aβ but with a level of final ThT fluorescence intensity that is about 40% of that observed for the apo‐Aβ. Additionally, in the presence of **HXE**, fibrillar aggregates are recovered. This may be related to the formation of a ternary species, [Cu(**XE**)(Im_Aβ_)]^+^, that was detected by UV‐Vis when using the Aβ_16_ fragment at similar concentration (Figure S13). The formation of the ternary species may leave to Aβ its ability to self‐assemble but to a slightly different morphology with distinct response to ThT. Note that the possibility of having inner‐filter effect has been ruled out based on the low absorptivity of the **HXE**‐based Cu^2+^ complexes in the dedicated wavelength range. The recovery of the kinetics parameters and fibrillar structure induced by **HXE** indicates that the ligand can modulate the Cu^2+^(Aβ_40_) aggregation pathway, deflecting it from the generation of soluble oligomeric species, widely described as the most neurotoxic forms, and promoting the formation of mature fibrils, which exhibit reduced cellular toxicity [137].

In presence of Zn^2+^, Aβ aggregation data similar to those reported in the literature were obtained, with a rapid nonsigmoidal process [24, 81, 133, 138]. The addition of **HXE** did not impact Zn^2+^(Aβ_40_) aggregation kinetics or resulting aggregates morphology, in line with a weaker to similar affinity for Zn^2+^ of the **HXE** *versus* the Aβ ligands (Table 5).

Kinetic curves of all replicates are shown in Figures S14, S15 and the respective kinetic parameters can be found in Table S6.

## 4 Conclusions

**FIGURE 16:**
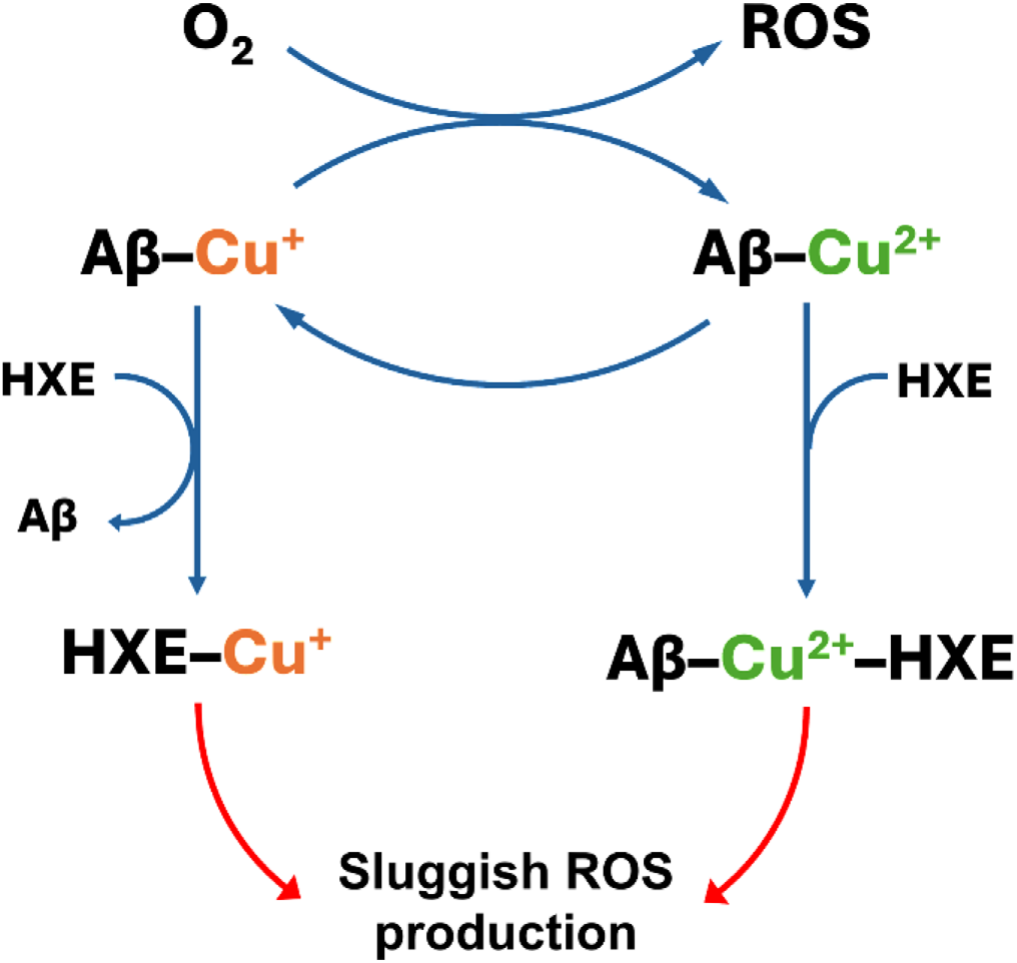
Overview of Cu⁺/Cu^2^⁺ chelation by **HXE** and its impact on Aβ(Cu)‐mediated ROS generation.

High extracellular levels of copper have been widely associated with the progression of AD [11]. In particular, the interaction between Cu ions and the Aβ peptide favors the formation of toxic aggregates and the generation of ROS due to the cycling between Cu⁺ and Cu^2^⁺ [27]. In this context, chelating agents have been proposed as a promising therapeutic approach to prevent this interaction [5, 32, 33, 34, 37, 40]. However, to date, no chelating agent has shown significant clinical efficacy [42, 51], with most being designed to specifically target Cu^2+^. In this work, we report the synthesis and characterization, both in the solid state and in solution, of a new 1‐methylimidazole‐containing thiosemicarbazone (**HXE**). Due to the presence of the biocompatible imidazole group, the compound shows a good solubility in aqueous medium and low toxicity, as demonstrated against HT‐22 mouse hippocampal neuronal cells. In addition, all pharmacokinetic parameters predicted *in silico* are within the expected values for compounds with the potential to cross the BBB. **HXE** forms 1:1 complexes in pseudo‐physiological solution with Cu^2+^, Cu^+^, and Zn^2+^, presenting the respective affinity constants of log *K*_app_ = 12.3, 8.7 and 5.0. These values indicate that the ligand is highly selective for both oxidation states of copper over zinc, a characteristic which is extremely important, given the high concentration of zinc present in the synaptic cleft. **HXE** has an affinity for Cu^2+^ approximately 100–1000 times greater than that of Aβ. Regarding Cu^+^, the difference is between one and two orders of magnitude in favor of our ligand. **HXE** is, in fact, very efficient in modulating Cu(Aβ) interactions, fully removing Cu^+^ from the peptide and, instead, forming a ternary [Cu(**XE**)(Im_Aβ_)]^+^ complex with Cu^2+^. Ternary complexes may be involved in an alternative mechanism of metal passivation, and their formation can be considered, in many respects, desirable. The group of Mi Hee Lim, for example, described ternary complexes of Cu^2+^(Aβ) with bidentate chemical regulators that can specifically modulate Cu‐induced Aβ aggregation [139, 140]. Consistent with the **HXE** properties demonstrated in this study (Figure 16), the ligand was able to significantly reduce Cu(Aβ)‐mediated ROS production, even in the presence of 10 equivalents of zinc. **HXE** also restored the half‐time (t_½_) of Cu^2+^(Aβ_40_) aggregation to levels comparable to that of the apo form of the peptide and induced the formation of fibrillar structures typical of those of apo‐Aβ aggregates.

Together, our results demonstrate that the new thiosemicarbazone **HXE** presents a promising profile to modulate Cu^+^(Aβ) and Cu^2+^(Aβ) interactions, thus mitigating Cu‐induced Aβ toxicity. In this sense, this work contributes not only to the understanding of the bioinorganic mechanisms of AD, but also to the development of ligands with novel structural motifs and a real therapeutic potential.

## Author Contributions

CH, NAR, CE: Conceptualization; BM, CE, CH: Writing, Formal analysis, Investigation, Review & editing; AG, MVC, AB, SML, JO, CK: Writing, Formal analysis, Investigation; NAR, CH: Resources.

## Conflicts of Interest

The authors declare no conflict of interest.

## Supporting information




**Supporting File 1**: chem70495‐sup‐0001‐SuppMat.pdf.

## Data Availability

The data that support the findings of this study are available from the corresponding author upon reasonable request.

## References

[1] J. Gale , E. Aizenman , “The Physiological and Pathophysiological Roles of Copper in the Nervous System,” European Journal of Neuroscience 60 (2024): 3505–3543. 38747014 10.1111/ejn.16370 PMC11491124

[2] E. I. Solomon , D. E. Heppner , E. M. Johnston , et al., “Copper Active Sites in Biology,” Chemical Reviews 114 (2014): 3659–3853. 24588098 10.1021/cr400327t PMC4040215

[3] G. Gromadzka , B. Tarnacka , A. Flaga , A. Adamczyk , “Copper Dyshomeostasis in Neurodegenerative Diseases—Therapeutic Implications,” International Journal of Molecular Sciences 21 (2020): 9259. 33291628 10.3390/ijms21239259 PMC7730516

[4] S. Lutsenko , S. Roy , P. Tsvetkov , “Mammalian Copper homeostasis: Physiological Roles and Molecular Mechanisms,” Physiological Reviews 105 (2025): 441–491. 39172219 10.1152/physrev.00011.2024 PMC11918410

[5] C. Esmieu , S. Hostachy , C. Hureau , “Cu(I) chelators: Useful Tools to Reveal and Control Cu(I) Homeostasis and Toxicity,” Coordination Chemistry Reviews 539 (2025): 216684.

[6] Z. Zhu , M. Song , J. Ren , L. Liang , G. Mao , M. Chen , “Copper Homeostasis and Cuproptosis in Central Nervous System Diseases,” Cell death & disease 15 (2024): 850. 39567497 10.1038/s41419-024-07206-3 PMC11579297

[7] R. Squitti , P. Faller , C. Hureau , A. Granzotto , A. R. White , K. P. Kepp , “Copper Imbalance in Alzheimer's Disease and Its Link With the Amyloid Hypothesis: Towards a Combined Clinical, Chemical, and Genetic Etiology,” Journal of Advanced Dielectrics 83 (2021): 23–41. 10.3233/JAD-201556 34219710

[8] A. Sabalic , V. Mei , G. Solinas , R. Madeddu , “The Role of Copper in Alzheimer's Disease Etiopathogenesis: An Updated Systematic Review,” Toxics 12 (2024): 755. 39453175 10.3390/toxics12100755 PMC11511397

[9] K. Marković , M. Cemazar , G. Sersa , R. Milačič , J. Ščančar , “Speciation of Copper in Human Serum Using Conjoint Liquid Chromatography on Short‐Bed Monolithic Disks With UV and Post Column ID‐ICP‐MS Detection,” Journal of Analytical Atomic Spectrometry 37 (2022): 1675–1686.

[10] Y. An , S. Li , X. Huang , X. Chen , H. Shan , M. Zhang , “The Role of Copper Homeostasis in Brain Disease,” International Journal of Molecular Sciences 23 (2022): 13850. 36430330 10.3390/ijms232213850 PMC9698384

[11] F. Liu , Z. Zhang , L. Zhang , et al., “Effect of metal ions on Alzheimer's disease,” Brain and Behavior 12 (2022): e2527. 35212185 10.1002/brb3.2527 PMC8933773

[12] N. Das , J. Raymick , S. Sarkar , “Role of metals in Alzheimer's disease,” Metabolic Brain Disease 36 (2021): 1627–1639. 34313926 10.1007/s11011-021-00765-w

[13] H. W. Ejaz , W. Wang , M. Lang , “Copper Toxicity Links to Pathogenesis of Alzheimer's Disease and Therapeutics Approaches,” International Journal of Molecular Sciences 21 (2020): 7660. 33081348 10.3390/ijms21207660 PMC7589751

[14] J. Everett , F. Lermyte , J. Brooks , et al., “Biogenic Metallic Elements in the Human Brain?,” Science Advances 7 (2021): eabf6707. 34108207 10.1126/sciadv.abf6707 PMC8189590

[15] L. M. Miller , Q. Wang , T. P. Telivala , R. J. Smith , A. Lanzirotti , J. Miklossy , “Synchrotron‐Based Infrared and X‐ray Imaging Shows Focalized Accumulation of Cu and Zn co‐localized With β‐amyloid Deposits in Alzheimer's Disease,” Journal of Structural Biology 155 (2006): 30–37. 16325427 10.1016/j.jsb.2005.09.004

[16] M. A. Lovell , J. D. Robertson , W. J. Teesdale , J. L. Campbell , W. R. Markesbery , “Copper, iron and zinc in Alzheimer's Disease Senile Plaques,” Journal of the Neurological Sciences 158 (1998): 47–52. 9667777 10.1016/s0022-510x(98)00092-6

[17] J.‐Y. Hur , “γ‐Secretase in Alzheimer's Disease,” Experimental & Molecular Medicine 54 (2022): 433–446. 35396575 10.1038/s12276-022-00754-8 PMC9076685

[18] N. Candelise , S. Scaricamazza , I. Salvatori , et al., “Protein Aggregation Landscape in Neurodegenerative Diseases: Clinical Relevance and Future Applications,” International Journal of Molecular Sciences 22 (2021): 6016. 34199513 10.3390/ijms22116016 PMC8199687

[19] D. S. Knopman , H. Amieva , R. C. Petersen , et al., “Alzheimer Disease,” Nature reviews Disease primers 7 (2021): 33. 10.1038/s41572-021-00269-y PMC8574196 33986301

[20] H. Hampel , J. Hardy , K. Blennow , et al., “The Amyloid‐β Pathway in Alzheimer's Disease,” Molecular Psychiatry 26 (2021): 5481–5503. 34456336 10.1038/s41380-021-01249-0 PMC8758495

[21] Y. Zhang , R. Thompson , H. Zhang , H. Xu , “APP Processing in Alzheimer's disease,” Molecular Brain 4 (2011): 3. 21214928 10.1186/1756-6606-4-3 PMC3022812

[22] A. Abelein , S. Ciofi‐Baffoni , C. Mörman , et al., “Molecular Structure of Cu(II)‐Bound Amyloid‐β Monomer Implicated in Inhibition of Peptide Self‐Assembly in Alzheimer's Disease,” JACS Au 2 (2022): 2571–2584. 36465548 10.1021/jacsau.2c00438 PMC9709942

[23] M. G. M. Weibull , S. Simonsen , C. R. Oksbjerg , M. K. Tiwari , L. Hemmingsen , “Effects of Cu(II) on the Aggregation of Amyloid‐β,” Journal of Biological Inorganic Chemistry 24 (2019): 1197–1215. 31602542 10.1007/s00775-019-01727-5

[24] M. Rana , A. K. Sharma , “Cu and Zn interactions With Aβ Peptides: Consequence of Coordination on Aggregation and Formation of Neurotoxic Soluble Aβ Oligomers,” Metallomics 11 (2019): 64–84. 30234208 10.1039/c8mt00203g

[25] F. Hane , Z. Leonenko , “Effect of Metals on Kinetic Pathways of Amyloid‐β Aggregation,” Biomolecules 4 (2014): 101–116. 24970207 10.3390/biom4010101 PMC4030978

[26] A. K. Sharma , S. T. Pavlova , J. Kim , J. Kim , L. M. Mirica , “The Effect of Cu2+ and Zn2+ on the Aβ42 Peptide Aggregation and Cellular Toxicity,” Metallomics 5 (2013): 1529. 23995980 10.1039/c3mt00161j PMC4060528

[27] C. Cheignon , M. Tomas , D. Bonnefont‐Rousselot , P. Faller , C. Hureau , F. Collin , “Oxidative Stress and the Amyloid Beta Peptide in Alzheimer's Disease,” Redox Biology 14 (2018): 450–464. 29080524 10.1016/j.redox.2017.10.014 PMC5680523

[28] C. Cheignon , M. Jones , E. Atrián‐Blasco , et al., “Identification of Key Structural features of the Elusive Cu–Aβ Complex that Generates ROS in Alzheimer's Disease,” Chemical Science 8 (2017): 5107–5118. 28970897 10.1039/c7sc00809k PMC5613283

[29] K. Terpstra , C. Gutiérrez , K. Gui , L. M. Mirica , “Donepezil and Memantine Derivatives for Dual‐Function and Prodrug Applications in Alzheimer's Disease,” Acs Chemical Neuroscience 16 (2025): 3591–3602. 40864225 10.1021/acschemneuro.5c00493 PMC12414536

[30] J. Yoo , J. Lee , B. Ahn , J. Han , M. H. Lim , “Multi‐target‐Directed Therapeutic Strategies for Alzheimer's Disease: Controlling Amyloid‐β Aggregation, Metal Ion Homeostasis, and Enzyme Inhibition,” Chemical Science 16 (2025): 2105–2135. 39810997 10.1039/d4sc06762b PMC11726323

[31] A. Cendron , M. Chianese , K. Zarzycki , et al., “Chelating Properties of N6O‐Donors Toward Cu(II) Ions: Speciation in Aqueous Environments and Catalytic Activity of the Dinuclear Complexes,” Molecules (Basel, Switzerland) 29 (2024): 5708. 39683868 10.3390/molecules29235708 PMC11643690

[32] T. Mazur , M. Malik , D. C. Bieńko , “The impact of Chelating Compounds on Cu2+, Fe2+/3+, and Zn2+ Ions in Alzheimer's Disease Treatment,” Journal of Inorganic Biochemistry 257 (2024): 112601. 38744143 10.1016/j.jinorgbio.2024.112601

[33] A. Gucký , S. Hamuľaková , “Targeting Biometals in Alzheimer's Disease With Metal Chelating Agents Including Coumarin Derivatives,” CNS Drugs 38 (2024): 507–532. 38829443 10.1007/s40263-024-01093-0 PMC11182807

[34] S. K. Singh , V. Balendra , A. A. Obaid , et al., “Copper‐Mediated Β‐Amyloid Toxicity And Its Chelation Therapy In Alzheimer's Disease,” Metallomics 14 (2022): mfac018. 35333348 10.1093/mtomcs/mfac018

[35] M. Rana , H.‐J. Cho , H. Arya , et al., “Azo‐Stilbene and Pyridine–Amine Hybrid Multifunctional Molecules to Target Metal‐Mediated Neurotoxicity and Amyloid‐β Aggregation in Alzheimer's Disease,” Inorganic Chemistry 61 (2022): 10294–10309. 35768324 10.1021/acs.inorgchem.2c00502

[36] M. Spiegel , T. Marino , M. Prejanò , N. Russo , “Antioxidant And Copper‐Chelating Power Of New Molecules Suggested As Multiple Target Agents Against Alzheimer's Disease. A theoretical comparative study,” Physical Chemistry Chemical Physics 24 (2022): 16353–16359. 35762619 10.1039/d2cp01918c

[37] K. D. Fasae , A. O. Abolaji , T. R. Faloye , et al., “Metallobiology And Therapeutic Chelation Of Biometals (Copper, Zinc And Iron) In Alzheimer's Disease: Limitations, And Current And Future Perspectives,” Journal of Trace Elements in Medicine and Biology 67 (2021): 126779. 34034029 10.1016/j.jtemb.2021.126779

[38] T. Storr , “Multifunctional Compounds For The Treatment Of Alzheimer's Disease,” Canadian Journal of Chemistry 99 (2021): 1–9.

[39] H.‐J. Cho , A. K. Sharma , Y. Zhang , M. L. Gross , L. M. Mirica , “A Multifunctional Chemical Agent as an Attenuator of Amyloid Burden and Neuroinflammation in Alzheimer's Disease,” Acs Chemical Neuroscience 11 (2020): 1471–1481. 32310630 10.1021/acschemneuro.0c00114 PMC7732605

[40] M. G. Savelieff , G. Nam , J. Kang , H. J. Lee , M. Lee , M. H. Lim , “Development of Multifunctional Molecules as Potential Therapeutic Candidates for Alzheimer's Disease, Parkinson's Disease, and Amyotrophic Lateral Sclerosis in the Last Decade,” Chem. Rev. 119 (2019): 1221–1322. 30095897 10.1021/acs.chemrev.8b00138

[41] V. Chaudhari , S. Bagwe‐Parab , H. S. Buttar , S. Gupta , A. Vora , G. Kaur , “Challenges and Opportunities of Metal Chelation Therapy in Trace Metals Overload‐Induced Alzheimer's Disease,” Neurotoxicity Research 41 (2023): 270–287. 36705861 10.1007/s12640-023-00634-7

[42] C. Esmieu , D. Guettas , A. Conte‐Daban , L. Sabater , P. Faller , C. Hureau , “Copper‐Targeting Approaches in Alzheimer's Disease: How To Improve the Fallouts Obtained From in Vitro Studies,” Inorganic Chemistry 58 (2019): 13509–13527. 31247877 10.1021/acs.inorgchem.9b00995

[43] C. Hureau , “Coordination Of Redox Active Metal Ions To The Amyloid Precursor Protein And To Amyloid‐Β Peptides Involved In Alzheimer Disease. Part 1: An overview,” Coordination Chemistry Reviews 256 (2012): 2164–2174.

[44] B. Alies , H. Eury , C. Bijani , L. Rechignat , P. Faller , C. Hureau , “pH‐Dependent Cu(II) Coordination to Amyloid‐β Peptide: Impact of Sequence Alterations, Including the H6R and D7N Familial Mutations,” Inorganic Chemistry 50 (2011): 11192–11201. 21980910 10.1021/ic201739n

[45] B. Alies , E. Renaglia , M. Rózga , W. Bal , P. Faller , C. Hureau , “Cu(II) Affinity for the Alzheimer's Peptide: Tyrosine Fluorescence Studies Revisited,” Analytical Chemistry 85 (2013): 1501–1508. 23249207 10.1021/ac302629u

[46] T. R. Young , A. Kirchner , A. G. Wedd , Z. Xiao , “An Integrated Study Of The Affinities Of The Aβ16 Peptide For Cu(I) And Cu(Ii): Implications For The Catalytic Production Of Reactive Oxygen Species†,” Metallomics 6 (2014): 505–517. 24493126 10.1039/c4mt00001c

[47] B. Alies , B. Badei , P. Faller , C. Hureau , “Reevaluation of Copper(I) Affinity for Amyloid‐β Peptides by Competition With Ferrozine—An Unusual Copper(I) Indicator,” Chemistry A European Journal 18 (2012): 1161–1167. 22189983 10.1002/chem.201102746

[48] N. Yako , T. R. Young , J. M. Cottam Jones , C. A. Hutton , A. G. Wedd , Z. Xiao , “Copper Binding And Redox Chemistry Of The Aβ16 Peptide And Its Variants: Insights Into Determinants Of Copper‐Dependent Reactivity,” Metallomics 9 (2017): 278–291. 28145544 10.1039/c6mt00299d

[49] Z. Xiao , L. Gottschlich , R. van der Meulen , S. R. Udagedara , A. G. Wedd , “Evaluation Of Quantitative Probes For Weaker Cu(I) Binding Sites Completes A Set Of Four Capable Of Detecting Cu(I) Affinities From Nanomolar To Attomolar†,” Metallomics 5 (2013): 501–513. 23579336 10.1039/c3mt00032j

[50] A. Conte‐Daban , B. Boff , A. Candido Matias , et al., “A Trishistidine Pseudopeptide With Ability to Remove Both CuΙ and CuΙΙ From the Amyloid‐β Peptide and to Stop the Associated ROS Formation,” Chemistry—A European Journal 23 (2017): 17078–17088. 28846165 10.1002/chem.201703429 PMC5714062

[51] A. Conte‐Daban , A. Day , P. Faller , C. Hureau , “How Zn Can Impede Cu Detoxification By Chelating Agents In Alzheimer's Disease: A Proof‐Of‐Concept Study,” Dalton Transactions 45 (2016): 15671–15678. 27711738 10.1039/c6dt02308h PMC5123634

[52] G. R., Pearson in Chemical Hardness (Berlin, Heidelberg: Springer, 1993): 1–10.

[53] E. Atrián‐Blasco , E. Cerrada , A. Conte‐Daban , et al., “Copper(I) Targeting In The Alzheimer's Disease Context: A First Example Using The Biocompatible Pta Ligand,” Metallomics 7 (2015): 1229–1232. 25926057 10.1039/c5mt00077g

[54] E. Atrián‐Blasco , E. Cerrada , P. Faller , M. Laguna , C. Hureau , “Role Of Pta In The Prevention Of Cu(Amyloid‐Β) Induced Ros Formation And Amyloid‐Β Oligomerisation In The Presence Of Zn,” Metallomics 11 (2019): 1154–1161. 31098605 10.1039/c9mt00011a PMC6588526

[55] K. P. Malikidogo , M. Drommi , E. Atrián‐Blasco , et al., “Ability of Azathiacyclen Ligands To Stop Cu(Aβ)‐Induced Production of Reactive Oxygen Species: [3N1S] Is the Right Donor Set,” Chemistry A European Journal 29 (2023): e202203667. 36606721 10.1002/chem.202203667

[56] C. Rulmont , J.‐L. Stigliani , C. Hureau , C. Esmieu , “Rationally Designed Cu(I) Ligand to Prevent CuAβ‐Generated ROS Production in the Alzheimer's Disease Context,” Inorganic Chemistry 63 (2024): 2340–2351. 38243896 10.1021/acs.inorgchem.3c02693

[57] A. Conte‐Daban , M. Beyler , R. Tripier , C. Hureau , “Kinetics Are Crucial When Targeting Copper Ions to Fight Alzheimer's Disease: An Illustration With Azamacrocyclic Ligands,” Chemistry—A European Journal 2018, 24, 8447–8452. 29611877 10.1002/chem.201801520

[58] A. Conte‐Daban , M. Beyler , R. Tripier , C. Hureau , “Corrigendum: Kinetics Are Crucial When Targeting Copper Ions to Fight Alzheimer's Disease: An Illustration With Azamacrocyclic Ligands,” Chemistry—A European Journal 24 (2018): 13058–13058. 30175501 10.1002/chem.201803841

[59] D. S. Cukierman , N. A. Rey , “Tridentate N‐Acylhydrazones as Moderate Ligands for the Potential Management of Cognitive Decline Associated With Metal‐Enhanced Neuroaggregopathies,” Frontiers Neurology (2022): 13, 10.3389/fneur.2022.828654. PMC8888665 35250832

[60] A. De Falco , G. C. Kincheski , E. Atrián‐Blasco , C. Hureau , S. T. Ferreira , N. A. Rey , “The Aroylhydrazone Inhhq Prevents Memory Impairment Induced By Alzheimer's‐Linked Amyloid‐Β Oligomers In Mice,” Behavioural Pharmacology 31 (2020): 738. 32773452 10.1097/FBP.0000000000000578

[61] D. S. Cukierman , E. Accardo , R. G. Gomes , et al., “Aroylhydrazones Constitute A Promising Class Of ‘Metal‐Protein Attenuating Compounds’ For The Treatment Of Alzheimer's Disease: A Proof‐Of‐Concept Based On The Study Of The Interactions Between Zinc(Ii) And Pyridine‐2‐Carboxaldehyde Isonicotinoyl Hydrazone,” Journal of Biological Inorganic Chemistry 23 (2018): 1227–1241. 30145655 10.1007/s00775-018-1606-0

[62] D. S. Cukierman , A. B. Pinheiro , S. L. P. Castiñeiras‐Filho , et al., “A Moderate Metal‐Binding Hydrazone Meets The Criteria For A Bioinorganic Approach Towards Parkinson's Disease: Therapeutic Potential, Blood‐Brain Barrier Crossing Evaluation And Preliminary Toxicological Studies,” Journal of Inorganic Biochemistry 170 (2017): 160–168. 28249224 10.1016/j.jinorgbio.2017.02.020

[63] R. A. Hauser‐Davis , L. V. de Freitas , D. S. Cukierman , et al., “Disruption of Zinc and Copper Interactions With Aβ(1–40) by a non‐toxic, Isoniazid‐Derived, Hydrazone: A Novel Biometal Homeostasis Restoring Agent in Alzheimer's Disease Therapy?†,” Metallomics 7 (2015): 743–747. 25860559 10.1039/c5mt00003c

[64] L. V. De Freitas , C. C. P. Da Silva , J. Ellena , L. A. S. Costa , N. A. Rey , “Structural and Vibrational Study of 8‐hydroxyquinoline‐2‐carboxaldehyde ISsonicotinoyl Hydrazone—A Potential Metal–Protein Attenuating Compound (MPAC) for the Treatment of Alzheimer's disease,” Spectrochimica Acta Part A: Molecular and Biomolecular Spectroscopy 116 (2013): 41–48. 23896296 10.1016/j.saa.2013.06.105

[65] B. M. Barbosa , D. C. Pires , A. Galvácsi , et al., “A New Cu^2+^ ‐Binding 1,3‐Benzodioxole‐Contaning *N* ‐Acylhydrazone Protects *Saccharomyces Cerevisiae* Cells From Oxidative Stress,” ChemistrySelect 10 (2025): e202405195.

[66] E. S. Helena , A. De Falco , D. S. Cukierman , A. Gioda , C. R. Gioda , N. A. Rey , “Cardiotoxicity and ROS Protection Assessment of three Structure‐Related N‐Acylhydrazones With Potential for the Treatment of Neurodegenerative Diseases,” Chemistry & Biodiversity 21 (2024): e202400356. 38353670 10.1002/cbdv.202400356

[67] A. Carvalho , B. M. Barbosa , J. S. Flores , et al., “New Mescaline‐Related N‐Acylhydrazone And Its Unsubstituted Benzoyl Derivative: Promising Metallophores For Copper‐Associated Deleterious Effects Relief In Alzheimer's Disease,” Journal of Inorganic Biochemistry 238 (2023): 112033. 36396525 10.1016/j.jinorgbio.2022.112033

[68] D. S. Cukierman , N. Bodnár , R. Diniz , L. Nagy , C. Kállay , N. A. Rey , “Full Equilibrium Picture in Aqueous Binary and Ternary Systems Involving Copper(II), 1‐Methylimidazole‐Containing Hydrazonic Ligands, and the 103–112 Human Prion Protein Fragment,” Inorganic Chemistry 61 (2022): 723–737. 34918515 10.1021/acs.inorgchem.1c03598

[69] D. S. Cukierman , D. F. Lázaro , P. Sacco , et al., “X1inh, An Improved Next‐Generation Affinity‐Optimized Hydrazonic Ligand, Attenuates Abnormal Copper(I)/Copper(Ii)‐Α‐Syn Interactions and Affects Protein Aggregation In A Cellular Model Of Synucleinopathy,” Dalton Transactions 49 (2020): 16252–16267. 32391542 10.1039/d0dt01138j

[70] A. Czylkowska , M. Pitucha , A. Raducka , et al., “Thiosemicarbazone‐Based Compounds: A Promising Scaffold for Developing Antibacterial, Antioxidant, and Anticancer Therapeutics,” Molecules (Basel, Switzerland) 30 (2024): 129. 39795184 10.3390/molecules30010129 PMC11721278

[71] E. J. Siddiqui , I. Azad , D. A. R. Khan , D. T. Khan , “THIOSEMICARBAZONE COMPLEXES AS VERSATILE MEDICINAL CHEMISTRY AGENTS: A REVIEW,” Journal of Drug Delivery and Therapeutics 9 (2019): 689–703.

[72] J. M. Wasielewska , K. Szostak , L. E. McInnes , et al., “Patient‐Derived Blood‐Brain Barrier Model for Screening Copper Bis(thiosemicarbazone) Complexes as Potential Therapeutics in Alzheimer's Disease,” Acs Chemical Neuroscience 15 (2024): 1432–1455. 38477556 10.1021/acschemneuro.3c00743

[73] B. Kaya , U. Acar Çevik , A. Karakaya , T. Ercetin , “Synthesis, Anticholinesterase and Antioxidant Activity of Thiosemicarbazone Derivatives,” Cumhuriyet Science Journal 45 (2024): 519–523.

[74] M. Varma , V. Ugale , J. Shaukat , et al., “Novel Alkyl‐Substituted 4‐Methoxy Benzaldehyde Thiosemicarbazones: Multi‐Target Directed Ligands For The Treatment Of Alzheimer's Disease,” European Journal of Pharmacology 957 (2023): 176028. 37657740 10.1016/j.ejphar.2023.176028

[75] B. Mavroidi , A. Kaminari , D. Matiadis , et al., “The Prophylactic and Multimodal Activity of Two Isatin Thiosemicarbazones Against Alzheimer's Disease In Vitro,” Brain Sciences 12 (2022): 806. 35741690 10.3390/brainsci12060806 PMC9221192

[76] S. Zaib , R. Munir , M. T. Younas , et al., “Hybrid Quinoline‐Thiosemicarbazone Therapeutics as a New Treatment Opportunity for Alzheimer's Disease‒Synthesis, In Vitro Cholinesterase Inhibitory Potential and Computational Modeling Analysis,” Molecules (Basel, Switzerland) 26 (2021): 6573. 34770983 10.3390/molecules26216573 PMC8587653

[77] M. Sagnou , B. Mavroidi , A. Kaminari , N. Boukos , M. Pelecanou , “Novel Isatin Thiosemicarbazone Derivatives as Potent Inhibitors of β‐Amyloid Peptide Aggregation and Toxicity,” Acs Chemical Neuroscience 11 (2020): 2266–2276. 32598129 10.1021/acschemneuro.0c00208

[78] D. Palanimuthu , R. Poon , S. Sahni , et al., “A Novel Class Of Thiosemicarbazones Show Multi‐Functional Activity For The Treatment Of Alzheimer's Disease,” European Journal of Medicinal Chemistry 139 (2017): 612–632. 28841514 10.1016/j.ejmech.2017.08.021

[79] D. S. Ranade , A. M. Bapat , S. N. Ramteke , et al., “Thiosemicarbazone Modification Of 3‐Acetyl Coumarin Inhibits Aβ Peptide Aggregation And Protect Against Aβ‐Induced Cytotoxicity,” European Journal of Medicinal Chemistry 121 (2016): 803–809. 26232353 10.1016/j.ejmech.2015.07.028

[80] L. M. F. Gomes , R. P. Vieira , M. R. Jones , et al., “8‐Hydroxyquinoline Schiff‐Base Compounds As Antioxidants And Modulators Of Copper‐Mediated Aβ Peptide Aggregation,” Journal of Inorganic Biochemistry 139 (2014): 106–116. 25019963 10.1016/j.jinorgbio.2014.04.011

[81] L. de Cremoux , E. Falcone , D. Schmitt , et al., “Modulation Of Aβ1–40 And Aβ4–40 Co‐Assembly By Zinc: Getting Closer To The Biological Reality,” Inorganic Chemistry Frontiers (2025), 10.1039/D5QI00850F.

[82] A. Conte‐Daban , V. Borghesani , S. Sayen , et al., “Link Between Affinity and Cu(II) Binding Sites to Amyloid‐β Peptides Evaluated by a New Water‐Soluble UV–Visible Ratiometric Dye With a Moderate Cu(II) Affinity,” Analytical Chemistry 89 (2017): 2155–2162. 28208266 10.1021/acs.analchem.6b04979 PMC5714188

[83] A. Conte‐Daban , V. Ambike , R. Guillot , N. Delsuc , C. Policar , C. Hureau , “A Metallo Pro‐Drug to Target Cu^II^ in the Context of Alzheimer's Disease,” Chemistry A European Journal 24 (2018): 5095–5099. 29334419 10.1002/chem.201706049 PMC6120673

[84] G. M. Sheldrick , “Crystal Structure Refinement With SHELXL,” Acta Cryst C 71 (2015): 3–8. 10.1107/S2053229614024218 PMC4294323 25567568

[85] H. R. Farias , J. M. O. Ramos , C. T. Griesang , et al., “LDL Exposure Disrupts Mitochondrial Function and Dynamics in a Hippocampal Neuronal Cell Line” Molecular Neurobiology 2025, 62, 6939–6950. 39302616 10.1007/s12035-024-04476-y

[86] T. Mosmann , “Rapid Colorimetric Assay For Cellular Growth And Survival: Application To Proliferation And Cytotoxicity Assays,” Journal of Immunological Methods 65 (1983): 55–63. 6606682 10.1016/0022-1759(83)90303-4

[87] H. M. Irving , M. G. Miles , L. D. Pettit , “A Study Of Some Problems In Determining The Stoicheiometric Proton Dissociation Constants Of Complexes By Potentiometric Titrations Using A Glass Electrode,” Analytica Chimica Acta 38 (1967): 475–488.

[88] P. Gans , A. Sabatini , A. Vacca , “SUPERQUAD: An Improved General Program For Computation Of Formation Constants From Potentiometric Data,” Journal of the Chemical Society, Dalton Transactions (1985): 1195–1200.

[89] D. J. Leggett , ed. Ed., Computational Methods for the Determination of Formation Constants, Springer US, Boston, MA, 1985., Computational Methods for the Determination of Formation Constants (Boston, MA: Springer US).

[90] , “ I. Puigdomenech (2006) HYDRA (Hydrochemical Equilibrium‐Constant Database) and MEDUSA (Make Equilibrium Diagrams Using Sophisticated Algorithms) Programs. Royal Institute of Technology, Stockholm.—References—Scientific Research Publishing,” can be found under https://www.scirp.org/reference/referencespapers?referenceid=1591507(accessed 21 August 2025), **n.d**.

[91] C. Esmieu , R. Balderrama‐Martínez‐Sotomayor , A. Conte‐Daban , O. Iranzo , C. Hureau , “Unexpected Trends in Copper Removal From Aβ Peptide: When Less Ligand Is Better and Zn Helps,” Inorganic Chemistry 60 (2021): 1248–1256. 33400522 10.1021/acs.inorgchem.0c03407

[92] A. Carvalho , K. C. Pougy , A. S. Pinheiro , D. S. Cukierman , N. A. Rey , “Differential Modulation of Copper(II) Interactions With the 18–22 Coordinating Amylin Fragment by the Geometric Isomers of a New Nicotinoyl Hydrazone: A First Study,” ACS Omega 10 (2025): 31115–31127. 40727756 10.1021/acsomega.5c04850 PMC12290932

[93] G. Palla , G. Predieri , P. Domiano , C. Vignali , W. Turner , “Conformational Behaviour And /Isomerization Of ‐Acyl And ‐Aroylhydrazones,” Tetrahedron 42 (1986): 3649–3654.

[94] A. V. Afonin , D. V. Pavlov , A. V. Albanov , A. G. Mal'kina , “Solvent‐Induced E/Z Isomerization Of 2‐(Furylmethylidene)‐1‐Hydrazinecarbothioamide: The N–H⋅⋅⋅O Intramolecular Hydrogen Bond As Promoting Factor,” Journal of Molecular Structure 1207 (2020): 127782.

[95] M. L. A. Temperini , M. R. Dos Santos , V. R. Paoli Monteiro , “Spectroscopic Study Of The Isomerization Of Z‐ To E‐Pyridine‐2‐Formyl Thiosemicarbazone,” Spectrochimica Acta Part A: Molecular and Biomolecular Spectroscopy 51 (1995): 1517–1524.

[96] C. A. Lipinski , F. Lombardo , B. W. Dominy , P. J. Feeney , “Experimental And Computational Approaches To Estimate Solubility And Permeability In Drug Discovery And Development Settings1,” Advanced Drug Delivery Reviews 46 (2001): 3–26. 11259830 10.1016/s0169-409x(00)00129-0

[97] P. D. Leeson , R. J. Young , “Molecular Property Design: Does Everyone Get It?,” Acs Medicinal Chemistry Letters 6 (2015): 722–725. 26191353 10.1021/acsmedchemlett.5b00157 PMC4499821

[98] H. Van De Waterbeemd , G. Camenisch , G. Folkers , J. R. Chretien , O. A. Raevsky , “Estimation of Blood‐Brain Barrier Crossing of Drugs Using Molecular Size and Shape, and H‐Bonding Descriptors,” Journal of Drug Targeting 6 (1998): 151–165. 9886238 10.3109/10611869808997889

[99] K. Palm , P. Stenberg , K. Luthman , “Polar Molecular Surface Properties Predict the Intestinal Absorption of Drugs in Humans,” Pharmaceutical Research 1 (1997): 568–571. 10.1023/a:1012188625088 9165525

[100] D. E. Clark , “Rapid Calculation Of Polar Molecular Surface Area And Its Application To The Prediction Of Transport Phenomena. 1. Prediction Of Intestinal Absorption,” Journal of Pharmaceutical Sciences 88 (1999): 807–814. 10430547 10.1021/js9804011

[101] H. Kadry , B. Noorani , L. Cucullo , “A Blood–Brain Barrier Overview On Structure, Function, Impairment, and Biomarkers Of Integrity,” Fluids and Barriers of the CNS 17 (2020): 69. 33208141 10.1186/s12987-020-00230-3 PMC7672931

[102] L. Yang , D. R. Powell , R. P. Houser , “Structural Variation In Copper(I) Complexes With Pyridylmethylamide Ligands: Structural Analysis With A New Four‐Coordinate Geometry Index,” Dalton Transactions (2007): 955–964. 17308676 10.1039/b617136b

[103] J. C. Mareque‐Rivas , R. Prabaharan , S. Parsons , “Quantifying The Relative Contribution Of Hydrogen Bonding And Hydrophobic Environments, And Coordinating Groups, In The Zinc(Ii)–Water Acidity By Synthetic Modelling Chemistry,” Dalton Transactions (2004): 1648–1655. 15252616 10.1039/b402084g

[104] N. Elgrishi , K. J. Rountree , B. D. McCarthy , E. S. Rountree , T. T. Eisenhart , J. L. Dempsey , “A Practical Beginner's Guide to Cyclic Voltammetry,” Journal of Chemical Education 95 (2018): 197–206.

[105] R. Kotuniak , W. Bal , “Kinetics Of Cu(Ii) Complexation By Atcun/Nts And Related Peptides: A Gold Mine Of Novel Ideas For Copper Biology,” Dalton Transactions 51 (2021): 14–26. 34816848 10.1039/d1dt02878b

[106] B. K. Maiti , N. Govil , T. Kundu , J. J. G. Moura , “Designed Metal‐ATCUN Derivatives: Redox‐ and Non‐redox‐Based Applications Relevant for Chemistry, Biology, and Medicine,” Iscience 23 (2020): 101792. 33294799 10.1016/j.isci.2020.101792 PMC7701195

[107] P. Gonzalez , K. Bossak , E. Stefaniak , et al., “N‐Terminal Cu‐Binding Motifs (Xxx‐Zzz‐His, Xxx‐His) and Their Derivatives: Chemistry, Biology and Medicinal Applications,” Chemistry A European J 24 (2018): 8029–8041. 10.1002/chem.201705398 PMC6152890 29336493

[108] R. Kotuniak , M. J. F. Strampraad , K. Bossak‐Ahmad , et al., “Key Intermediate Species Reveal the Copper(II)‐Exchange Pathway in Biorelevant ATCUN/NTS Complexes,” Angewandte Chemie International Edition 59 (2020): 11234–11239. 32267054 10.1002/anie.202004264 PMC7383912

[109] A. Noormägi , T. Golubeva , E. Berntsson , S. K. T. S. Wärmländer , V. Tõugu , P. Palumaa , “Direct Competition of ATCUN Peptides With Human Serum Albumin for Copper(II) Ions Determined by LC‐ICP MS,” ACS Omega 8 (2023): 33912–33919. 37744839 10.1021/acsomega.3c04649 PMC10515390

[110] K. Bossak‐Ahmad , T. Frączyk , W. Bal , S. C. Drew , “The Sub‐picomolar Cu^2+^ Dissociation Constant of Human Serum Albumin,” Chembiochem 21 (2020): 331–334. 31298451 10.1002/cbic.201900435

[111] L. A. Yatsunyk , A. C. Rosenzweig , “Cu(I) Binding and Transfer by the N Terminus of the Wilson Disease Protein,” Journal of Biological Chemistry 282 (2007): 8622–8631. 17229731 10.1074/jbc.M609533200

[112] Z. Xiao , P. S. Donnelly , M. Zimmermann , A. G. Wedd , “Transfer of Copper Between Bis(thiosemicarbazone) Ligands and Intracellular Copper‐Binding Proteins. Insights Into Mechanisms of Copper Uptake and Hypoxia Selectivity,” Inorganic Chemistry 47 (2008): 4338–4347. 18412332 10.1021/ic702440e

[113] G. Arena , E. Rizzarelli , “Zn2+ Interaction With Amyloid‐B: Affinity and Speciation,” Molecules (Basel, Switzerland) 24 (2019): 2796. 31370315 10.3390/molecules24152796 PMC6695645

[114] S. Noël , S. Bustos Rodriguez , S. Sayen , E. Guillon , P. Faller , C. Hureau , “Use of a New Water‐Soluble Zn Sensor to Determine Zn Affinity for the Amyloid‐β Peptide and Relevant Mutants†,” Metallomics 6 (2014): 1220–1222. 24652294 10.1039/c4mt00016a

[115] I. Zawisza , M. Rózga , W. Bal , “Affinity of Copper and Zinc Ions to Proteins and Peptides Related to Neurodegenerative Conditions (Aβ, APP, α‐Synuclein, PrP),” Coordination Chemistry Reviews 256 (2012): 2297–2307.

[116] D. Kahra , M. Kovermann , P. Wittung‐Stafshede , “The C‐Terminus of Human Copper Importer Ctr1 Acts as a Binding Site and Transfers Copper to Atox1,” Biophysical Journal 110 (2016): 95–102. 26745413 10.1016/j.bpj.2015.11.016 PMC4805863

[117] C. Esmieu , G. Ferrand , V. Borghesani , C. Hureau , “Impact of N‐Truncated Aβ Peptides on Cu‐ and Cu(Aβ)‐Generated ROS: CuI Matters!,” Chemistry—A European Journal 27 (2021): 1777–1786. 33058356 10.1002/chem.202003949

[118] C. Hureau , V. Balland , Y. Coppel , P. L. Solari , E. Fonda , P. Faller , “Importance Of Dynamical Processes In The Coordination Chemistry And Redox Conversion Of Copper Amyloid‐Β Complexes,” Journal of Biological Inorganic Chemistry 14 (2009): 995–1000. 19618220 10.1007/s00775-009-0570-0

[119] S. Chassaing , F. Collin , P. Dorlet , J. Gout , C. Hureau , P. Faller , “Copper And Heme‐Mediated Abeta Toxicity: Redox Chemistry, Abeta Oxidations and anti‐ROS Compounds,” Current Topics in Medicinal Chemistry 12 (2012): 2573–2595. 23339309 10.2174/1568026611212220011

[120] V. Borghesani , B. Alies , C. Hureau , “Cu(Ii) Binding To Various Forms Of Amyloid‐Β Peptides. Are they friends or foes?,” European Journal of Inorganic Chemistry 2018 (2018): 7–15. 30186035 10.1002/ejic.201700776 PMC6120674

[121] J. T. Pedersen , S. W. Chen , C. B. Borg , et al., “Amyloid‐β and α‐Synuclein Decrease the Level of Metal‐Catalyzed Reactive Oxygen Species by Radical Scavenging and Redox Silencing,” Journal of the American Chemical Society 138 (2016): 3966–3969. 26967463 10.1021/jacs.5b13577 PMC4827876

[122] D. Jiang , X. Li , L. Liu , G. B. Yagnik , F. Zhou , “Reaction Rates and Mechanism of the Ascorbic Acid Oxidation by Molecular Oxygen Facilitated by Cu(II)‐Containing Amyloid‐β Complexes and Aggregates,” Journal of Physical Chemistry B 114 (2010): 4896–4903. 20302320 10.1021/jp9095375 PMC2878184

[123] H. LeVine , Methods in Enzymology (Academic Press, 1999): 274–284. 10.1016/s0076-6879(99)09020-5 10507030

[124] C. Xue , T. Y. Lin , D. Chang , Z. Guo , “Thioflavin T As An Amyloid Dye: Fibril Quantification, Optimal Concentration And Effect On Aggregation,” Royal Society Open Science 4 (2017): 160696. 28280572 10.1098/rsos.160696 PMC5319338

[125] K. Gade Malmos , L. M. Blancas‐Mejia , B. Weber , et al., “ThT 101: A Primer on the use of Thioflavin T to Investigate Amyloid Formation,” Amyloid 24 (2017): 1–16. 10.1080/13506129.2017.1304905 28393556

[126] S. Noël , S. Cadet , E. Gras , C. Hureau , “The Benzazole Scaffold: A Swat to Combat Alzheimer's Disease,” Chem. Soc. Rev. 42 (2013): 7747–7762. 23793644 10.1039/c3cs60086f

[127] Y. Li , S. Awasthi , L. Bryan , et al., “Fluorescence‐Based Monitoring of Early‐Stage Aggregation of Amyloid‐β, Amylin Peptide, Tau, and α‐Synuclein Proteins,” Acs Chemical Neuroscience 15 (2024): 3113–3123. 39150403 10.1021/acschemneuro.4c00097 PMC11378287

[128] P. Faller , C. Hureau , O. Berthoumieu , “Role of Metal Ions in the Self‐Assembly of the Alzheimer's Amyloid‐β Peptide,” Inorganic Chemistry 52 (2013): 12193–12206. 23607830 10.1021/ic4003059

[129] O. N. Antzutkin , “Amyloidosis Of Alzheimer's Aβ Peptides: Solid‐State Nuclear Magnetic Resonance, Electron Paramagnetic Resonance, Transmission Electron Microscopy, Scanning Transmission Electron Microscopy And Atomic Force Microscopy Studies,” Magnetic Resonance in Chemistry 42 (2004): 231–246. 14745804 10.1002/mrc.1341

[130] M. Bartolini , M. Naldi , J. Fiori , et al., “Kinetic Characterization Of Amyloid‐Beta 1–42 Aggregation With A Multimethodological Approach,” Analytical Biochemistry 414 (2011): 215–225. 21435333 10.1016/j.ab.2011.03.020

[131] S. J. Wood , B. Maleeff , T. Hart , R. Wetzel , “Physical, Morphological and Functional Differences Between pH 5.8 and 7.4 Aggregates of the Alzheimer's Amyloid Peptide A β,” Journal of Molecular Biology 256 (1996): 870–877. 8601838 10.1006/jmbi.1996.0133

[132] E. Stefaniak , E. Atrian‐Blasco , W. Goch , L. Sabater , C. Hureau , W. Bal , “The Aggregation Pattern of Aβ1–40 is Altered by the Presence of N‐Truncated Aβ4–40 and/or CuII in a Similar Way Through Ionic Interactions,” Chemistry—A European Journal 27 (2021): 2798–2809. 33207022 10.1002/chem.202004484

[133] C. Cheignon , F. Collin , L. Sabater , C. Hureau , “Oxidative Damages on the Alzheimer's Related‐Aβ Peptide Alters Its Ability to Assemble,” Antioxidants 12 (2023): 472. 36830030 10.3390/antiox12020472 PMC9951946

[134] G. Vázquez , A. B. Caballero , J. Kokinda , A. Hijano , R. Sabaté , P. Gamez , “Copper, Dityrosine Cross‐Links And Amyloid‐Β Aggregation,” Journal of Biological Inorganic Chemistry 24 (2019): 1217–1229. 31667594 10.1007/s00775-019-01734-6

[135] F. Attanasio , P. D. Bona , S. Cataldo , et al., “Copper(Ii) And Zinc(Ii) Dependent Effects On Aβ42 Aggregation: A CD, Th‐T and SFM study,” New Journal of Chemistry 37 (2013): 1206–1215.

[136] E. Atrián‐Blasco , A. Conte‐Daban , C. Hureau , “Mutual Interference Of Cu And Zn Ions In Alzheimer's Disease: Perspectives At The Molecular Level,” Dalton Transactions 46 (2017): 12750–12759. 28937157 10.1039/c7dt01344b PMC5656098

[137] M. Tolar , J. Hey , A. Power , S. Abushakra , “Neurotoxic Soluble Amyloid Oligomers Drive Alzheimer's Pathogenesis and Represent a Clinically Validated Target for Slowing Disease Progression,” IJMS 22 (2021): 6355. 34198582 10.3390/ijms22126355 PMC8231952

[138] C. J. Matheou , N. D. Younan , J. H. Viles , “The Rapid Exchange of Zinc2+ Enables Trace Levels to Profoundly Influence Amyloid‐β Misfolding and Dominates Assembly Outcomes in Cu2+/Zn2+ Mixtures,” Journal of Molecular Biology 428 (2016): 2832–2846. 27320389 10.1016/j.jmb.2016.05.017

[139] S. Hong , Y. K. Go , J. S. Derrick , et al., “Advanced Electron Paramagnetic Resonance Studies of a Ternary Complex of Copper, Amyloid‐β, and a Chemical Regulator,” Inorganic Chemistry 57 (2018): 12665–12670. 30239184 10.1021/acs.inorgchem.8b01824

[140] M. W. Beck , J. S. Derrick , R. A. Kerr , et al., “Structure‐Mechanism‐Based Engineering of Chemical Regulators Targeting Distinct Pathological Factors in Alzheimer's Disease,” Nature Communications 7 (2016): 13115. 10.1038/ncomms13115 PMC5065625 27734843

