# Supplementary material for: Evaluation of a New Methylimidazole‐Containing Thiosemicarbazone as a Cu+/Cu2+‐Targeting Ligand in the Context of Alzheimer's Disease
Source: Chemistry. 2025 Nov 28;32(1):e02754. doi: 10.1002/chem.202502754 (PMC12759177; doi:10.1002/chem.202502754)
Supplement: Supplementary file 1 — Supporting File 1: chem70495‐sup‐0001‐SuppMat.pdf. [file CHEM-32-e02754-s001.pdf]

Evaluation of a New Methylimidazole-Containing Thiosemicarbazone as a Cu^{+}/Cu^{2+}-Targeting Ligand in the Context of Alzheimer's Disease

Barbara Marinho Barbosa, Charlène Esmieu*Antal Galvácsi,
Mariana Viana Costa, Adèle Brison, Sonia M. Ladeira, Jade de Oliveira,
Csilla Kállay, Christelle Hureau*, Nicolás A. Rey*

Supplementary Information

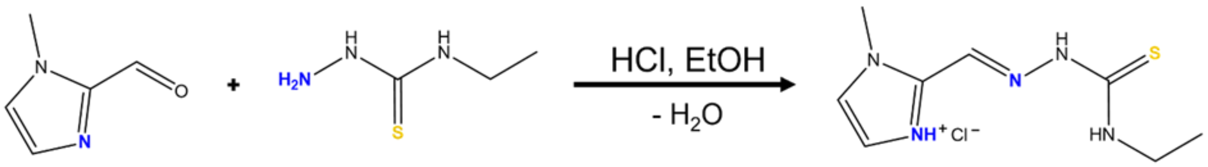

Fig. S1. Reactional scheme for the synthesis of **HXE**,HCl.

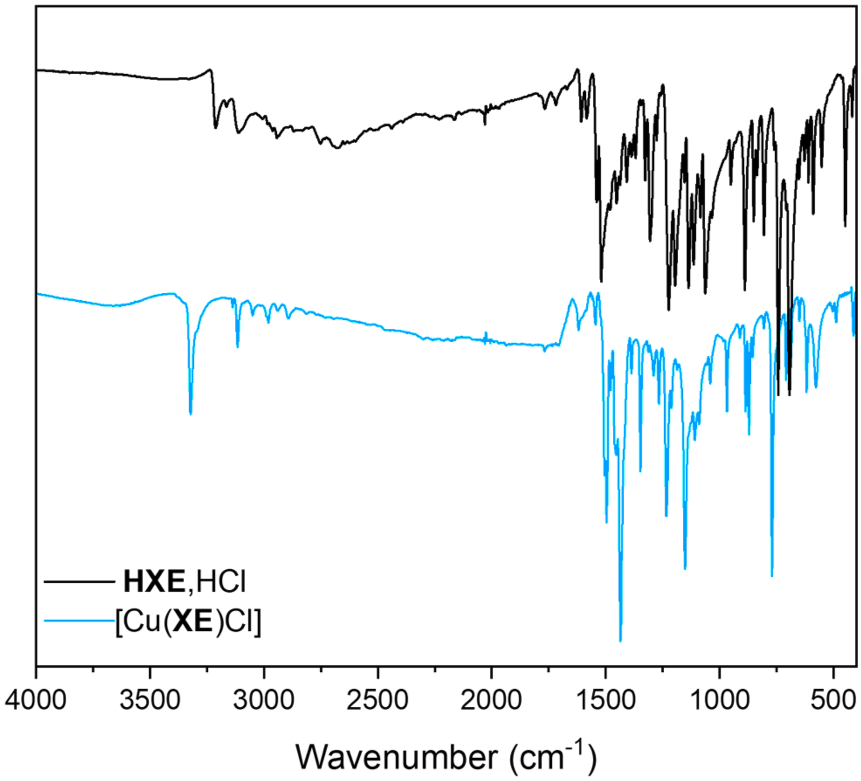

Fig. S2. Comparison of ATR infrared spectra of free ligand, **HXE**,HCl, and its copper complex [Cu(**XE**)Cl] at room temperature.

Table S1. Crystal, data collection and refinement parameters for **HXE**,HCl.

**Empirical formula**	**$C_8H_{14}ClN_5S$**
**Molecular weight (g mol^{-1})**	247.75
**Temperature (K)**	100(2)
**Crystal system**	Monoclinic
**Space group**	$P2_1/c$
**a (Å)**	12.3014(3)
**b (Å)**	7.21140(10)
**c (Å)**	14.2964(3)
**α (°)**	90
**β (°)**	114.997(2)
**γ (°)**	90
**Volume (Å^3)**	1149.44(4)
Z	4
**Density (mg.mm^{-3})**	1.432
**Crystal size (mm)**	0.20 × 0.14 × 0.06
**Independent reflections/unique**	21663 / 2476 [R(int) = 0.0402]
**Data/restraints/parameters**	2476/6/152
**"Goodness of fit on F^2"**	1.075
**Final R indexes [I≥2σ (I)]**	R1 = 0.0308, wR2 = 0.0811
**Final R indexes (all data)**	R1 = 0.0316, wR2 = 0.0817

Table S2. Selected geometric parameters for **HXE**,HCl.

**Bond distance (Å)**		**Bond angle (°)**	
**C5–N3**	1.2862(18)	**C4–C5–N3**	115.94(12)
**N3–N4**	1.3525(16)	**C5–N3–N4**	117.44(11)
**N4–C6**	1.3757(18)	**N3–N4–C6**	118.71(11)
**C6–N5**	1.3319(18)	**N4–C6–S1**	117.85(10)
**C6–S1**	1.6795(14)	**N4–C6–N5**	116.05(12)
		**S1–C6–N5**	126.10(11)

Table S3. H-bonding parameters for **HXE**,HCl.

D–H···A	D–H (Å)	H···A (Å)	D···A (Å)	D–H···A (°)
**N2–H···Cl**	0.883	2.253	3.1252	172.40
**N5–H···Cl**	0.852	2.512	3.3071	155.29
**N4–H···Cl*ⁱ***	0.892	2.373	3.2391	166.03

Symmetry code: *i* = (x, 3/2-y, 1/2+z)

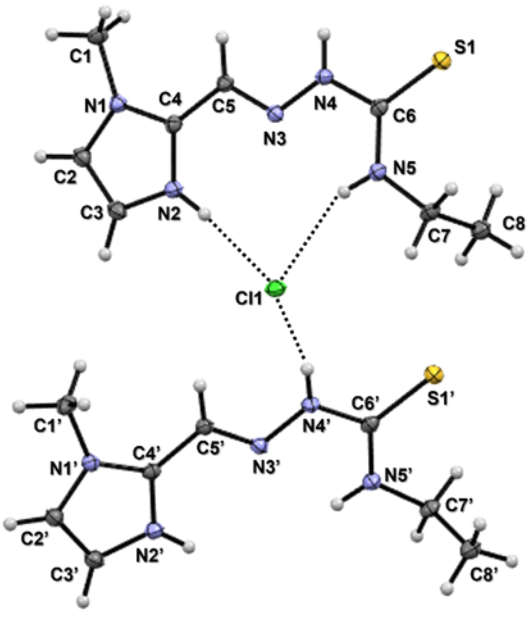

Fig. S3. H-bond interactions for **HXE**,HCl.

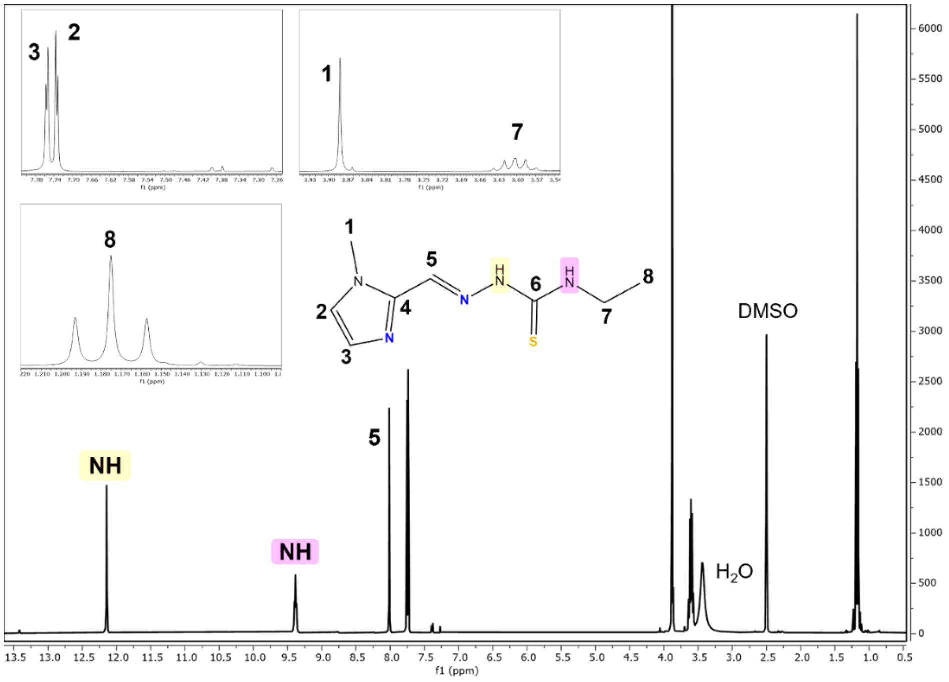

Fig. S4. ^{1}H NMR spectrum (400 MHz) of **HXE**,HCl in DMSO-d_6 at room temperature. $\delta_{\mathbf{HXE}}$: 1.18 (*t*, 3H), 3.60 (*m*, 2H), 3.88 (*s*, 3H), 7.73 (*d*, 1H, ^{3}J = 1.9), 7.76 (*d*, 1H, ^{3}J= 1.9), 8.01 (*d*, 1H), 9.28 (*t*, 1H), 12.14 (*s*, 1H).

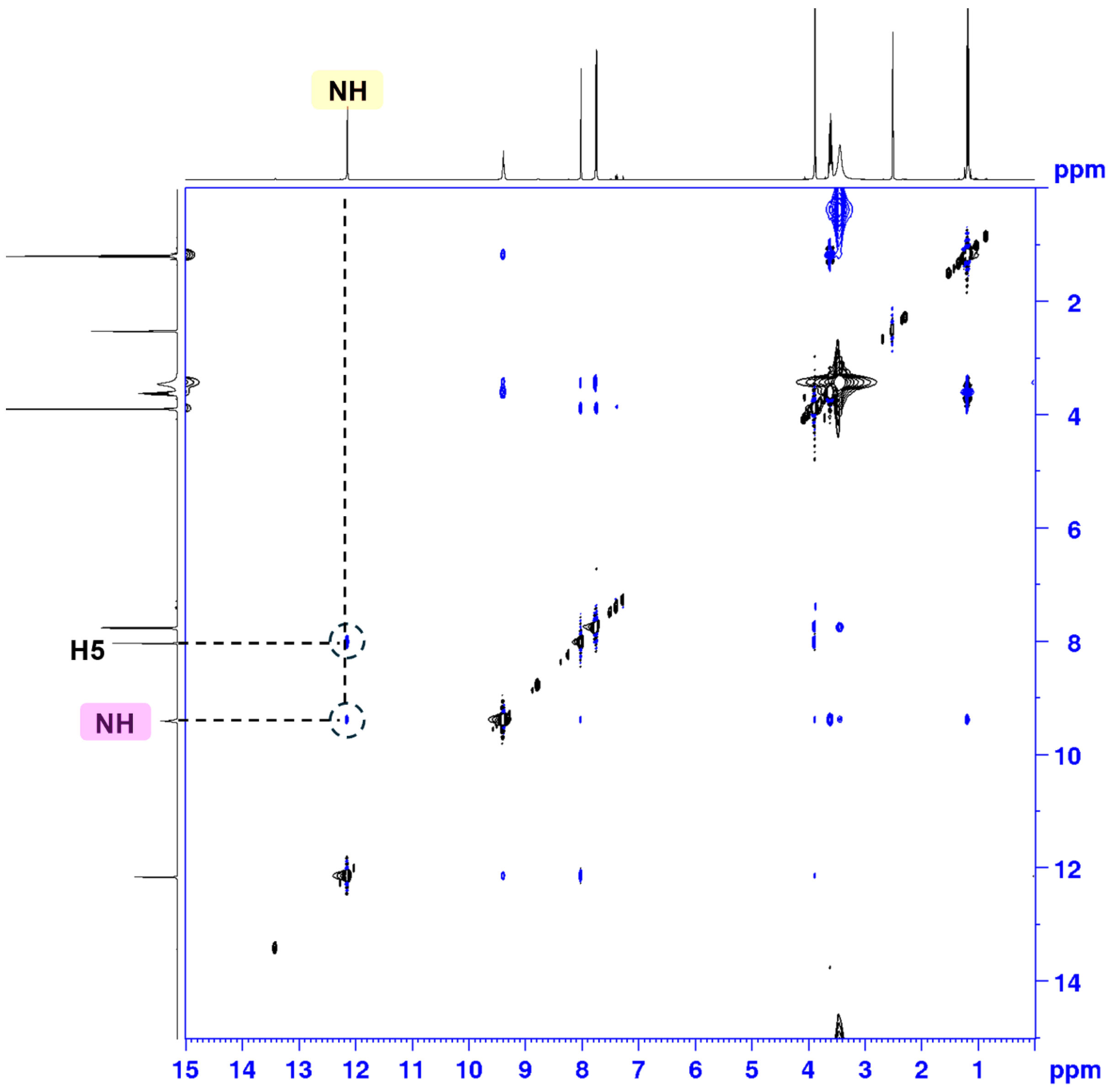

Fig. S5. NOESY contour plot (400 MHz) of **HXE**,HCl in DMSO-d_6 at room temperature.

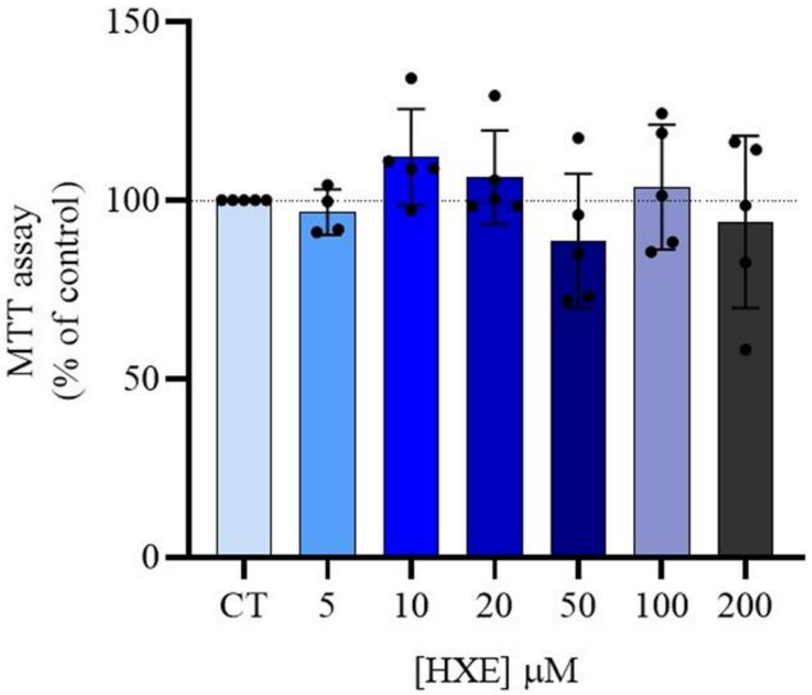

Fig. S6. Exposure to **HXE** did not affect the viability of HT-22 cells. Viability was assessed by MTT assay, and data are expressed as mean ± SEM. Statistical analysis was performed using one-way ANOVA test.

Table S4. Crystal, data collection and refinement parameters for [Cu(**XE**)Cl].

**Empirical formula**	$\mathbf{C_8H_{12}ClCuN_5S}$
**Molecular weight (g mol^{-1})**	309.29
**Temperature (K)**	100(2)
**Crystal system**	Orthorhombic
**Space group**	Pbca
**a (Å)**	14.3497(2)
**b (Å)**	8.80770(10)
**c (Å)**	18.6036(3)
**α (°)**	90
**β (°)**	90
**γ (°)**	90
**Volume (Å^3)**	2351.27(6)
Z	8
**Density (mg.mm^{-3})**	1.747
**Crystal size (mm)**	0.20 × 0.14 × 0.04
**Independent reflections/unique**	37846 / 2544 [R(int) = 0.0472]
**Data/restraints/parameters**	2544/0/151
**“Goodness of fit on F^2”**	1.110
**Final R indexes [I≥2σ (I)]**	R1 = 0.0328, wR2 = 0.0943
**Final R indexes (all data)**	R1 = 0.0351, wR2 = 0.0958

Table S5. Selected geometric parameters for [Cu(**XE**)Cl].

**Bond distance (Å)**		**Bond angle (°)**	
**Cu–N2**	1.996(2)	**N2–Cu–N3**	81.11(8)
**Cu–N3**	1.996(2)	**N2–Cu–S1**	164.68(6)
**Cu–S1**	2.2477(7)	**N2–Cu–Cl**	95.64(6)
**Cu-Cl**	2.2444(7)	**N3–Cu–S1**	83.58(6)
**N4−C6**	1.330(3)	**N3–Cu–Cl**	175.99(6)
**C6−S1**	1.755(2)	**S1–Cu–Cl**	99.66(3)

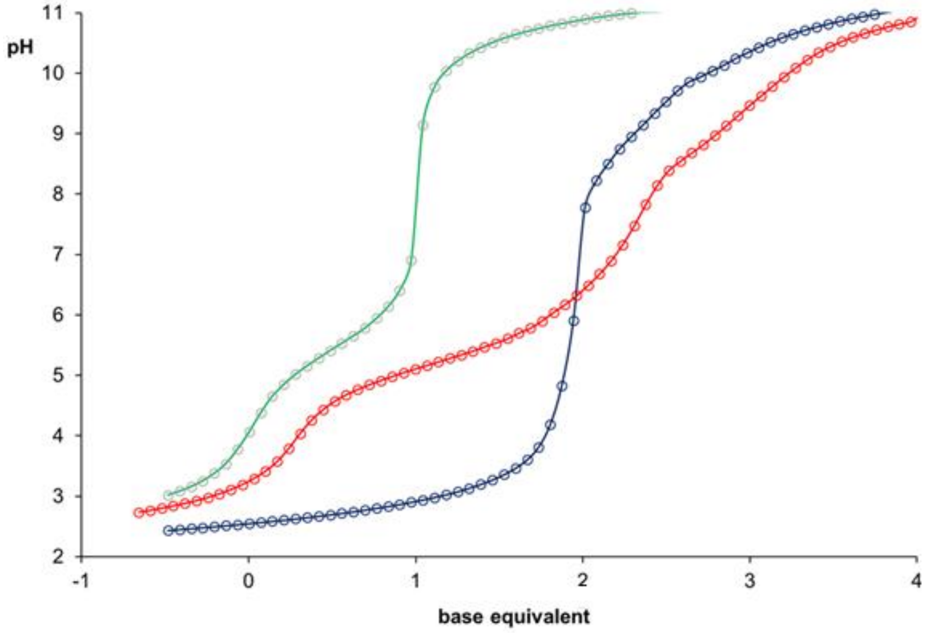

Fig. S7. Potentiometric titration curves of **HXE** (green) and the Cu^{2+}–**HXE** (blue) and Zn^{2+}–**HXE** (red) equimolar systems. [**HXE**] = 2.0 mM. The fitting parameters are $2.1\cdot\times 10^{-3}$ and $1.7\cdot\times 10^{-3}$, respectively.

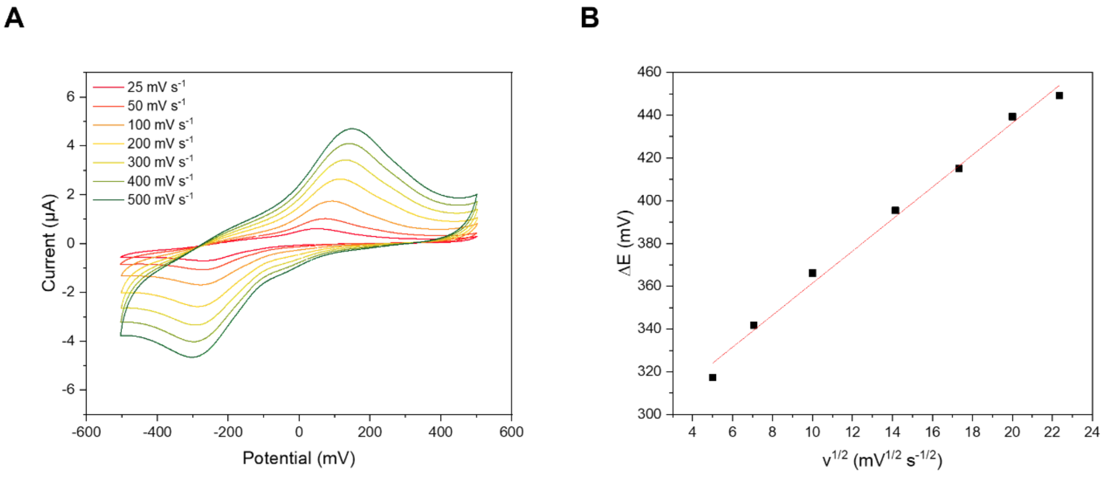

Fig. S8. (**A**) Voltammograms of $[Cu(\mathbf{XE})(H_2O)]^+$ at different scan rates and (**B**) ΔE dependence as function of the square-root of the scan rate. $[Cu^{2+}]$ = 90 μM and [**HXE**] = 100 μM in 50 mM HEPES pH 7.4. Electrodes' set employed: glassy carbon (WE) platinum wire (CE) and saturated calomel electrode (RE).

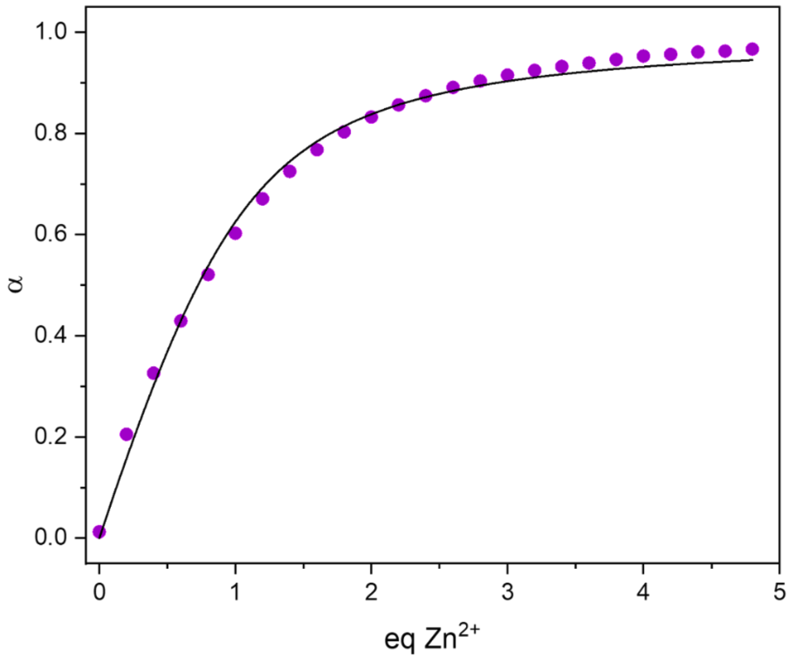

Fig. S9. Progression of $[Zn(\mathbf{XE})(H_2O)_x]^+$ formation as function of the number of equivalents of metal ion and theoretical curve (best fit). [**HXE**] = 50 µM in 50 mM HEPES pH 7.4.

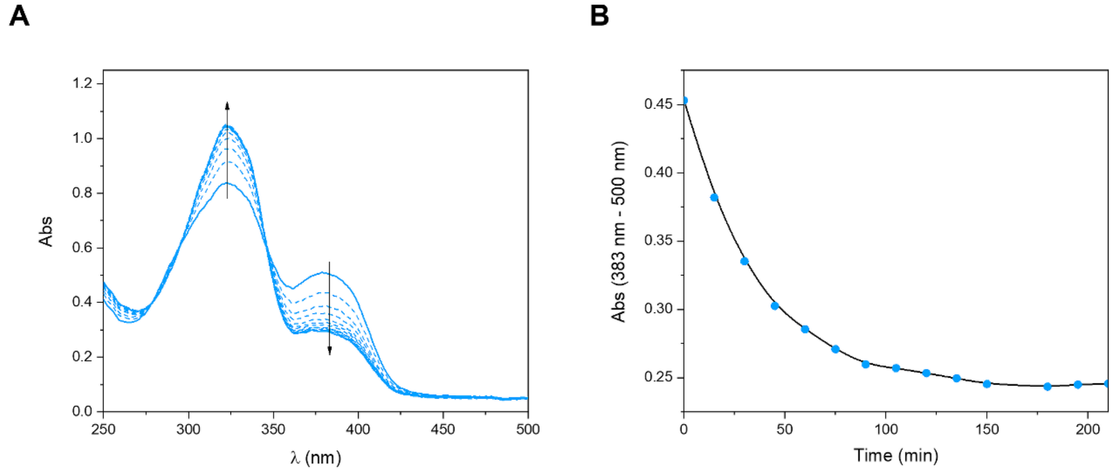

Fig. S10. Left: UV-Vis spectra of $[Cu(\mathbf{XE})(H_2O)]^+$ solution with the addition of 1 equivalent of competitor over time. **Right:** Kinectic curve of the dissossiation of $[Cu(\mathbf{XE})(H_2O)]^+$ complex by following the absorbance at 383 nm as function of time. [GGH] = [**HXE**] = 50 µM, $[Cu^{2+}]$ = 45 µM in 50 mM HEPES pH 7.4.

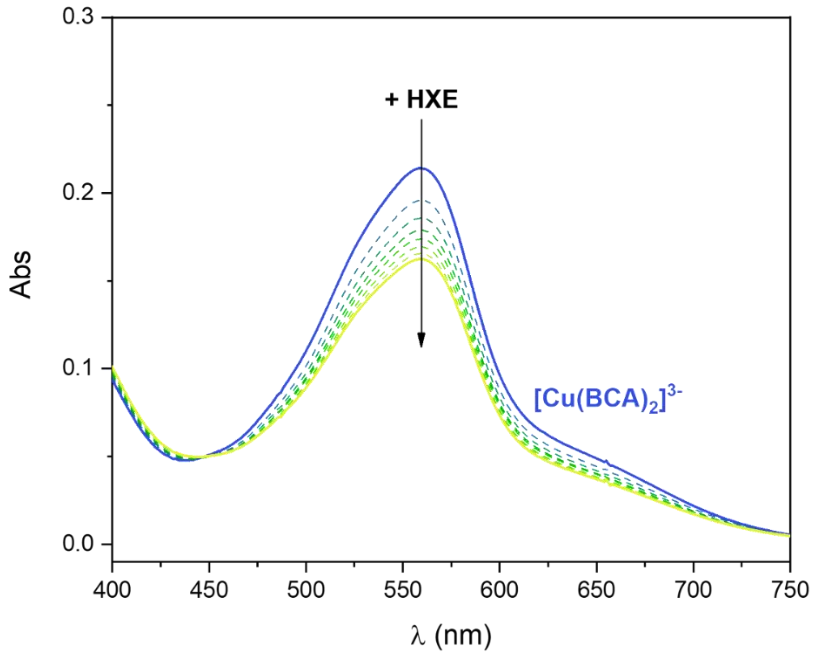

Fig. S11. Resulting spectra after consecutive additions of **HXE** (0.5 eq each addition) in a $[Cu(BCA)_2]^{3-}$ solution (50 mM HEPES pH 7.4).

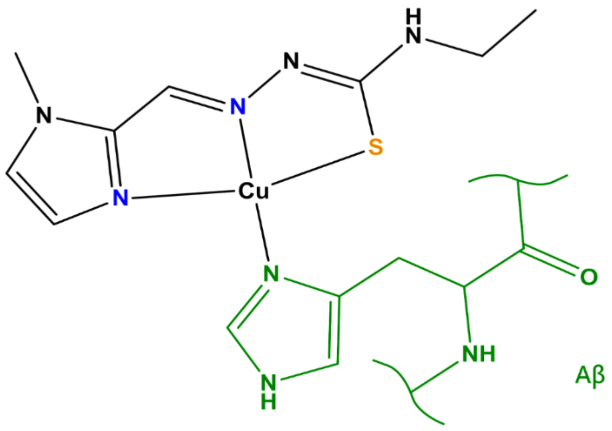

Fig. S12. Structural scheme for the ternary complex $[Cu(\mathbf{XE})(Im_{A\beta})]^{+}$.

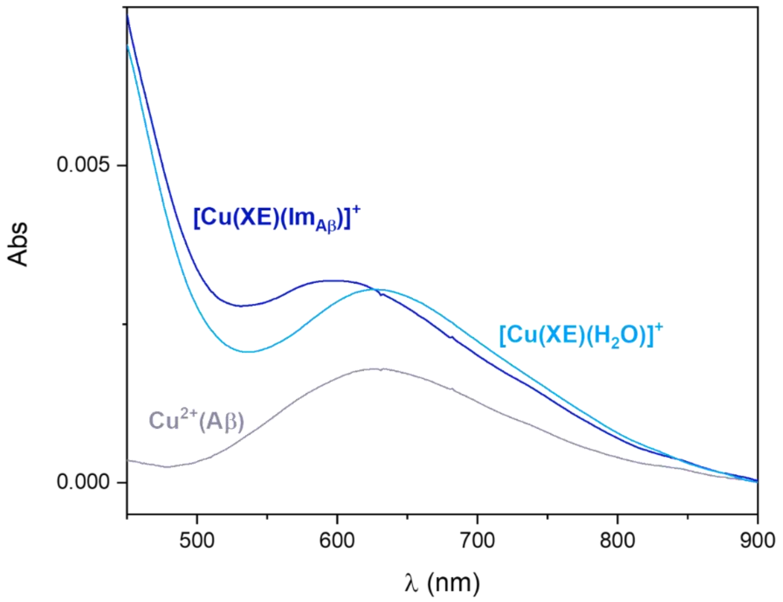

Fig. S13. *d-d* bands for the binary complexes {Cu^{2+}($A\beta_{16}$) and [Cu(**XE**)(H_2O)]$^+$} and the ternary complex [Cu(**XE**)($Im_{A\beta}$)]$^+$. [**HXE**] = [$A\beta_{16}$] = 24 µM, [Cu^{2+}] = 20 µM in 50 mM HEPES pH 7.4.

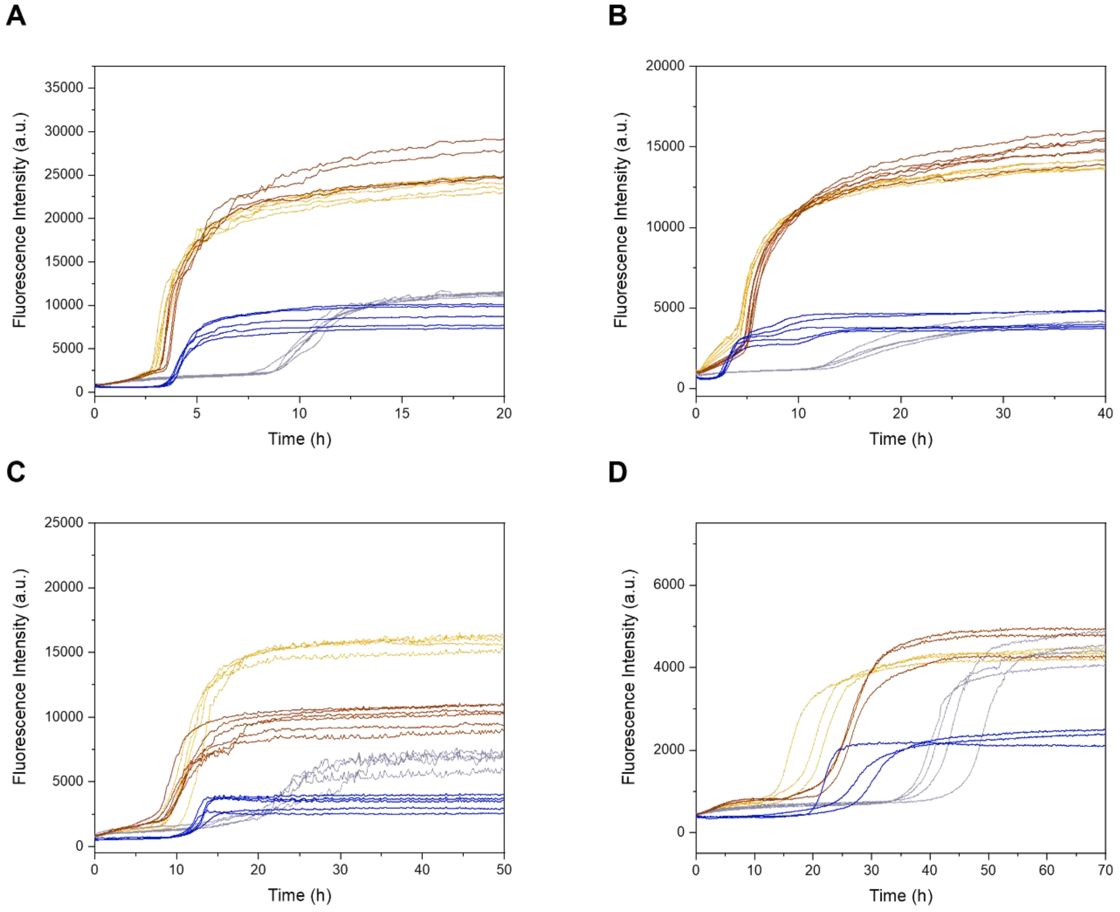

Fig. S14. Replicates of $A\beta_{40}$ aggregation experiment in the presence of Cu^{2+}. $A\beta_{40}$ (yellow), Cu^{2+}($A\beta_{40}$) (grey), $A\beta_{40}$ + **HXE** (brown) and Cu^{2+}($A\beta_{40}$) + **HXE** (blue) in 100 mM HEPES pH 7.4.
[Aβ] = [**HXE**] = 20 µM, [Cu^{2+}] = 18 µM, [ThT] = 10 µM and [EDTA] = 20 nM.

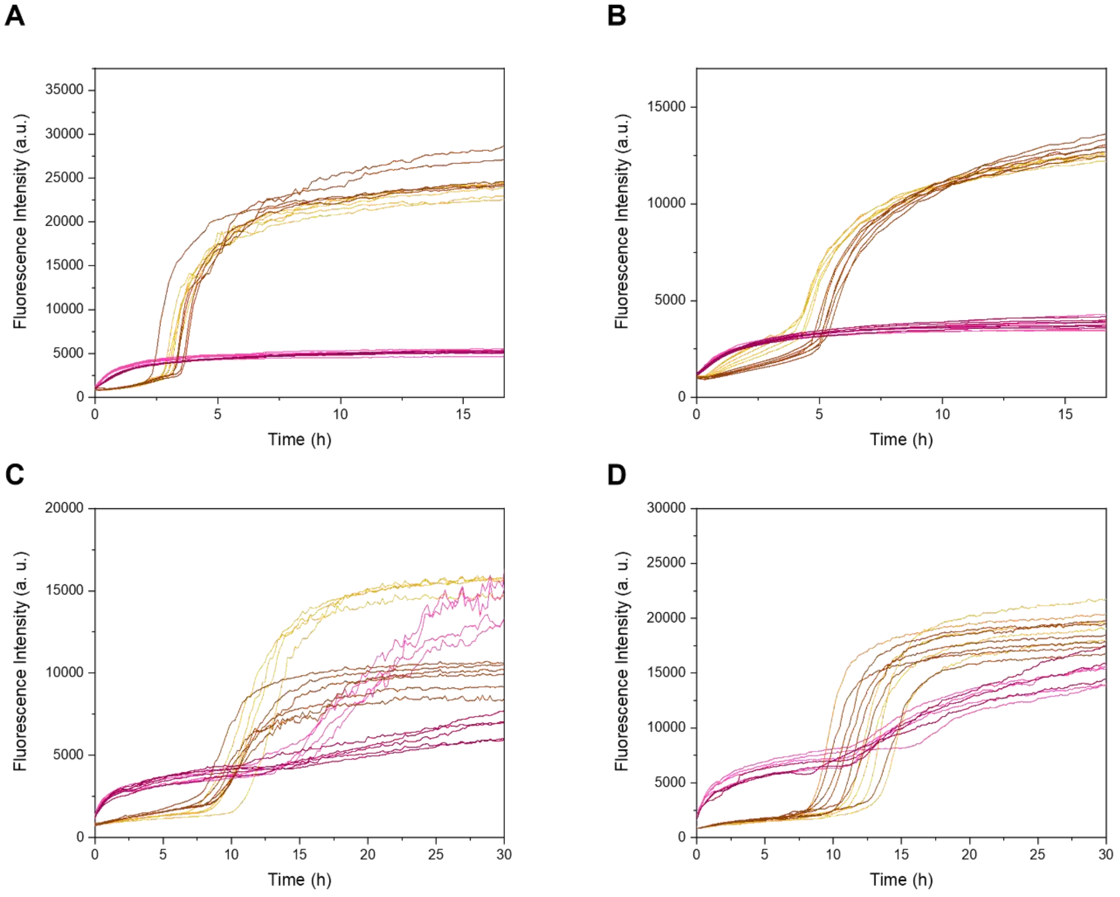

Fig. S15. Replicates of Aβ_{40} aggregation experiment in the presence of Zn^{2+}. Aβ_{40} (yellow), Zn^{2+}(Aβ_{40}) (violet), Aβ_{40} + **HXE** (brown) and Zn^{2+}(Aβ_{40}) + **HXE** (pink) in 100 mM HEPES pH 7.4. [Aβ] = [**HXE**] = 20 μM, [Zn^{2+}] = 18 μM, [ThT] = 10 μM and [EDTA] = 20 nM.

Table S6. Kinetic parameters of Aβ_{40} aggregation in the presence or absence of Cu^{2+} and **HXE**.

	**$t_{1/2}$ normalized**				**F_{max} normalized**			
	**A**	**B**	**C**	**D**	**A**	**B**	**C**	**D**
**Aβ + HXE**	1.14	1.18	0.90	1.33	1.09	1.12	0.63	1.08
**Cu^{2+}(Aβ_{40})**	2.71	3.12	1.97	1.95	0.51	0.27	0.45	1.17
**Cu^{2+}(Aβ_{40}) + HXE**	1.11	0.67	1.02	1.36	0.33	0.29	0.20	0.52
